# Targeting Bayes factors with direct-path non-equilibrium thermodynamic integration

**DOI:** 10.1007/s00180-017-0721-7

**Published:** 2017-03-14

**Authors:** Marco Grzegorczyk, Andrej Aderhold, Dirk Husmeier

**Affiliations:** 1 Johann Bernoulli Institute (JBI), Groningen University, Groningen, The Netherlands; 2 School of Mathematics and Statistics, Glasgow University, Glasgow, UK

**Keywords:** Bayesian inference, Marginal likelihood, Temperature ladder, Variance reduction, Jarzynski’s theorem, Benchmark studies, Biopathway

## Abstract

Thermodynamic integration (TI) for computing marginal likelihoods is based on an inverse annealing path from the prior to the posterior distribution. In many cases, the resulting estimator suffers from high variability, which particularly stems from the prior regime. When comparing complex models with differences in a comparatively small number of parameters, intrinsic errors from sampling fluctuations may outweigh the differences in the log marginal likelihood estimates. In the present article, we propose a TI scheme that directly targets the log Bayes factor. The method is based on a modified annealing path between the posterior distributions of the two models compared, which systematically avoids the high variance prior regime. We combine this scheme with the concept of non-equilibrium TI to minimise discretisation errors from numerical integration. Results obtained on Bayesian regression models applied to standard benchmark data, and a complex hierarchical model applied to biopathway inference, demonstrate a significant reduction in estimator variance over state-of-the-art TI methods.

## Introduction

A central quantity in Bayesian statistics is the marginal likelihood

1 $$\begin{aligned} p(D|{\mathcal {M}}) = \int p(D,{\varvec{\theta }}|{\mathcal {M}}) d{\varvec{\theta }} = \int p(D|{\varvec{\theta }},{\mathcal {M}}) p({\varvec{\theta }}|{\mathcal {M}}) d{\varvec{\theta }} \end{aligned}$$

where $D$ are the data, and ${\mathcal {M}}$ represents a given statistical model with parameter vector ${\varvec{\theta }}$. The difficulty in practically computing the marginal likelihood is exemplified by considering the Monte Carlo sum

2 $$\begin{aligned} X \; = \; \frac{1}{M} \sum _{i=1}^M p(D|{\varvec{\theta }}_i,{\mathcal {M}}) \end{aligned}$$

where $\{\theta _i\}$ is an iid sample from $p({\varvec{\theta }}|{\mathcal {M}})$. Under fairly general regularity conditions the estimator *X* converges almost surely to $p(D|{\mathcal {M}})$, by the strong law of large numbers, and is asymptotically efficient, with asymptotic variance $C/\sqrt{N}$ (where *N* is the size of $D$), by the central limit theorem. However, even for modestly complex systems, the constant in the numerator, *C*, can reach exorbitant magnitudes, rendering the scheme not viable for practical applications. The practical shortcomings of a variety of alternative numerical methods, like the harmonic mean estimator (Gelfand and Dey 1994), bridge sampling (Gelman and Meng 1998), or Chib’s method (Chib and Jeliazkov 2001), have been discussed in the statistics and machine learning literature (e.g. Murphy (2012)). The most widely used and robust method appears to be thermodynamic integration (TI). This method was originally proposed by Kirkwood (1935) and further developed in statistical physics for the mathematically equivalent problem of computing free energies; see e.g. Schlitter (1991) and Schlitter and Husmeier (1992). Gelman and Meng (1998) adapted TI to the computation of marginal likelihoods, Lartillot and Philippe (2006) demonstrated the application of TI to complex systems, and Friel and Pettitt (2008) and Calderhead and Girolami (2009) popularised TI more widely in the statistics community by demonstrating a computationally powerful combination with parallel tempering (Earl and Deem 2005).

Thermodynamic integration is based on an integral of the expected log likelihood along an inverse annealing path from the prior to the posterior distribution. The resulting estimator typically suffers from high variability, which particularly stems from the parameter prior regime. When comparing complex models with differences in a comparatively small number of parameters, these intrinsic errors from sampling fluctuations may outweigh the differences in the log marginal likelihood estimates. The objective of the present study is to explore the scope for variance reduction by directly targeting the log Bayes factor via a modified transition path between the two models such that the high-variance prior domain is avoided. This idea is not new. In statistical physics it is well known (Schlitter 1991; Schlitter and Husmeier 1992) that applying TI to the computation of a reaction free energy, which is mathematically equivalent to the log Bayes factor, is computationally more efficient than the separate computation of the standard free energies for the two reaction states involved (educt versus product states); the latter is mathematically equivalent to the difference of the log marginal likelihoods of two statistical models to be compared. Also in the statistics literature, the direct targeting of the log Bayes factor has been discussed before. For instance, path sampling (Gelman and Meng 1998) and annealed importance sampling (Neal 2001) have been conceived in a way to allow the direct computation of the ratio of two partition functions, $Z_1$ and $Z_2$, associated with two models ${\mathcal {M}}_1$ and ${\mathcal {M}}_2$. However, in the work of Neal (2001), $Z_1$ is set to the normalisation factor of the prior distribution, and the method thus reduces to the computation of the log marginal likelihood.^1^ Gelman and Meng (1998) do consider a direct comparison between two alternative models: a homoscedastic versus a heteroscedastic linear regression model. Rather than computing the Bayes factor, the authors apply their path sampling approach to infer the posterior distribution of the entire spectrum of intermediate models. While this is a more ambitious approach than model selection with Bayes factors, it will be computationally onerous beyond the one-dimensional regime considered in their example.

To the best of our knowledge, the present article presents the first systematic study of the variance reduction that can be achieved with a thermodynamic integration path that directly targets the log Bayes factor by transiting between the posterior distributions of the two models involved. The mathematical exposition and implementation of this scheme is combined with a comprehensive comparative performance assessment based on a set of standard benchmark data to quantify the improvement in variance reduction, accuracy and computationally efficiency that can be achieved over state-of-the-art established TI methods, in particular the recent improvement proposed by Friel et al. (2014).

This article is organised as follows. In Sect. 2 we provide a brief rationale for targeting Bayes factors directly rather than indirectly via the marginal likelihood. Section 3.1 reviews standard thermodynamic integration. In Sect. 3.2 we discuss a modified numerical integration and sampling scheme from statistical physics, termed non-equilibrium TI (NETI), to reduce numerical discretisation errors. Section 3.3 describes NETI-DIFF, the proposed new TI scheme along an alternative integration path between two posterior distributions. Sections 3.4, 3.5 describe practical numerical implementations based on Metropolis-Hastings and Gibbs sampling, and Sect. 3.6 proposes a new improved inverse temperature ladder. Section 4 provides an overview of a set of benchmark problems on which we have evaluated the methods, and Sect. 5 presents our empirical findings. We conclude this article in Sect. 6 with a discussion, a comparison with the controlled thermodynamic integral of Oates et al. (2016), and an outlook on future work.

## Rationale

Consider two alternative models, ${\mathcal {M}}_1$ and ${\mathcal {M}}_2$, and define $E_i= -\log p(D|{\varvec{\theta }},{\mathcal {M}}_i)$, the negative log likelihood of model *i*. Further, define the log likelihood ratio $\varDelta E= E_2-E_1$, the negative unnormalised log posterior ${\tilde{E}}_i= E_i+\log p({\varvec{\theta }}|{\mathcal {M}}_i)$, the negative log posterior ratio $\varDelta {\tilde{E}}= {\tilde{E}}_2-{\tilde{E}}_1$, and let $\langle \ldots \rangle _{i}$ denote the posterior average with respect to the posterior distribution $({\varvec{\theta }}|D,{\mathcal {M}}_i)$. We can then adapt Jarzynski’s theorem from statistical physics (Híjar and Zárate 2010) to show that

3 $$\begin{aligned} p(D|{\mathcal {M}}_i) = \Big \langle \exp (E_i[{\varvec{\theta }}])\Big \rangle _i^{-1}, \quad \quad \frac{p(D|{\mathcal {M}}_2)}{p(D|{\mathcal {M}}_1)} = \Big \langle \exp (-\varDelta {\tilde{E}}[{\varvec{\theta }}])\Big \rangle _1 \end{aligned}$$

A proof is given in the Appendix. In real applications with non-trivial models, the negative log likelihood is typically in the order of a two to three digit figure, which when put into the argument of the exponential function will lead to an astronomically large number. An estimator aiming to approximate $p(D|{\mathcal {M}}_i)= \langle \exp (E_i[{\varvec{\theta }}])\rangle _i^{-1}$ from a limited sample drawn from the posterior distribution will inevitably suffer from substantial variation. For nested models or models with sufficient parameter overlap, on the other hand, $\varDelta {\tilde{E}}({\varvec{\theta }})$ will typically be small, $|\varDelta {\tilde{E}}({\varvec{\theta }})| \ll \min \{|E_1({\varvec{\theta }})|,|E_2({\varvec{\theta }})|\}$. We can therefore reduce the intrinsic estimation uncertainty considerably by computing the Bayes factor directly rather than indirectly via two separate marginal likelihood estimations.

## Methodology

### Thermodynamic integration for marginal likelihoods

Thermodynamic integration is based on an inverse annealing path from the prior to the posterior distribution, and computing the expectation of the log likelihood with respect to the following annealed posterior distributions at inverse temperatures $\tau \in [0,1]$:

4 $$\begin{aligned} p_{\tau }({\varvec{\theta }}|D,{\mathcal {M}})= & {} \frac{1}{Z(D|\tau ,{\mathcal {M}})} p(D|{\varvec{\theta }},{\mathcal {M}})^{\tau } p({\varvec{\theta }}|{\mathcal {M}}), \nonumber \\ Z(D|\tau ,{\mathcal {M}})= & {} \int p(D|{\varvec{\theta }},{\mathcal {M}})^{\tau } p({\varvec{\theta }}|{\mathcal {M}}) d{\varvec{\theta }} \end{aligned}$$

Taking the derivative of $\log Z(D|\tau ,{\mathcal {M}})$ gives:

5 $$\begin{aligned} \frac{d}{d\tau } \log Z(D|\tau ,{\mathcal {M}})= & {} \frac{1}{Z(D|\tau ,{\mathcal {M}})} \frac{d}{d\tau } Z(D|\tau ,{\mathcal {M}})\nonumber \\= & {} \frac{1}{Z(D|\tau ,{\mathcal {M}})} \int \frac{d}{d\tau } p(D|{\varvec{\theta }},{\mathcal {M}})^{\tau } p({\varvec{\theta }}|{\mathcal {M}}) d{\varvec{\theta }} \nonumber \\= & {} \int \log p(D|{\varvec{\theta }},{\mathcal {M}}) \frac{ p(D|{\varvec{\theta }},{\mathcal {M}})^{\tau } p({\varvec{\theta }}|{\mathcal {M}})}{Z(D|\tau ,{\mathcal {M}})} d{\varvec{\theta }}\nonumber \\= & {} \int p_{\tau }({\varvec{\theta }}|D,{\mathcal {M}}) \log p(D|{\varvec{\theta }},{\mathcal {M}}) d{\varvec{\theta }} \nonumber \\= & {} {\mathbb {E}}_{\tau }\Big [ \log p(D|{\varvec{\theta }},{\mathcal {M}}) \Big ] \end{aligned}$$

From Eq. (5) we get:

6 $$\begin{aligned} \log p(D|{\mathcal {M}})= & {} \log Z(D|\tau =1) - \log Z(D|\tau =0) \nonumber \\= & {} \int _0^1 \frac{d}{d \tau }\log Z(D|\tau ) d \tau = \int _0^1 {\mathbb {E}}_{\tau }\Big [\log p(D|{\varvec{\theta }},{\mathcal {M}})\Big ] d\tau \end{aligned}$$

This one-dimensional integral can be solved numerically, e.g. with the trapezoid rule:

7 $$\begin{aligned} \log (p(D|{\mathcal {M}})) \approx \sum _{k=2}^K \frac{\tau _k-\tau _{k-1}}{2} \left\{ {\mathbb {E}}_{\tau _k}\big [\log p(D|{\varvec{\theta }},{\mathcal {M}})\big ] + {\mathbb {E}}_{\tau _{k-1}}\big [\log p(D|{\varvec{\theta }},{\mathcal {M}}) \big ] \right\} \end{aligned}$$

Some care has to be taken with respect to the choice of discretisation points $\tau _k, k=\{0,1,2,\ldots ,K\}$, as the major contributions to the integral usually come from a small region around $\tau \rightarrow 0$. This motivates the form

8 $$\begin{aligned} \tau _k = \left( \frac{k-1}{K-1}\right) ^{\alpha }; \quad k \in \{1,2,\ldots ,K\} \end{aligned}$$

for $\alpha > 1$. Theoretical results for the optimal choice of $\alpha$ can be found in Schlitter (1991), but require knowledge that is usually not available in practice (like the functional dependence of ${\mathbb {E}}_{\tau }[\log p(D|{\varvec{\theta }},{\mathcal {M}})]$ on $\tau$). In practice, $\alpha =5$ is widely used, as e.g. in Friel et al. (2014), and we have used this value in the present study. A potentially numerically more stable alternative was proposed in Friel et al. (2014). The authors show that:

9 $$\begin{aligned} \frac{d}{d\tau } \left\{ {\mathbb {E}}_{\tau }[\log (p(D|{\varvec{\theta }},{\mathcal {M}}))] \right\} _{\tau } = {\mathbb {V}}_{\tau }(\log (p(D|{\varvec{\theta }},{\mathcal {M}})) \end{aligned}$$

where ${\mathbb {V}}_{\tau }(.)$ is the variance w.r.t. the power posterior in Eq. (4). The second derivative of ${\mathbb {E}}_{\tau }[\log (p(D|{\varvec{\theta }},{\mathcal {M}}))]$ at a point $\tau \in [\tau _{k-1},\tau _{k}]$ can then be approximated by the difference quotient of the first derivative of ${\mathbb {E}}_{\tau }[\log p(D|{\varvec{\theta }},{\mathcal {M}})]$ Eq. (9):

$$\begin{aligned} \frac{d^2}{dt^2} \left\{ {\mathbb {E}}_{t}\left[ \log (p(D|{\varvec{\theta }},{\mathcal {M}}))\right] \right\} _{t=\tau } \approx \frac{{\mathbb {V}}_{\tau _{k}}(\log (p(D|{\varvec{\theta }},{\mathcal {M}}))-{\mathbb {V}}_{\tau _{k-1}}(\log (p(D|{\varvec{\theta }},{\mathcal {M}}))}{\tau _{k}-\tau _{k-1}} \end{aligned}$$

Friel et al. (2014) then employ the corrected trapezoid rule^2^ to compute each sub-integral $\int _{\tau _{k-1}}^{\tau _{k}}{\mathbb {E}}_{\tau }[\log (p(D|{\varvec{\theta }},{\mathcal {M}}))] d\tau$. This yields:

10 $$\begin{aligned} \log (p(D|{\mathcal {M}}))= & {} \int _{0}^{1} {\mathbb {E}}_{\tau } \big [ \log p(D|{\varvec{\theta }},{\mathcal {M}}) \big ] d \tau = \sum _{k=2}^K \int _{\tau _{k-1}}^{\tau _k} {\mathbb {E}}_{\tau } \big [ \log p(D|{\varvec{\theta }},{\mathcal {M}}) \big ] d \tau \nonumber \\\approx & {} \sum _{k=2}^K \frac{\tau _k-\tau _{k-1}}{2} \Bigg \{ {\mathbb {E}}_{\tau _k}\big [\log p(D|{\varvec{\theta }},{\mathcal {M}})\big ] + {\mathbb {E}}_{\tau _{k-1}}\big [\log p(D|{\varvec{\theta }},{\mathcal {M}}) \big ] \Bigg \} \nonumber \\&-\sum _{k=2}^K \frac{(\tau _k-\tau _{k-1})^2}{12} \Big \{ {\mathbb {V}}_{\tau _k}\big [\log p(D|{\varvec{\theta }},{\mathcal {M}})\big ] - {\mathbb {V}}_{\tau _{k-1}}\big [\log p(D|{\varvec{\theta }},{\mathcal {M}}) \big ] \Big \}\nonumber \\ \end{aligned}$$

### Nonequilibrium thermodynamic integration

The computation of the expectation values ${\mathbb {E}}_{\tau _k}\big [\log p(D|{\varvec{\theta }},{\mathcal {M}})\big ]$ is expensive and limits the number of discretisation points *K* that can be practically applied. An alternative scheme we use in the present work is to approximate

11 $$\begin{aligned} \log p(D|{\mathcal {M}})= & {} \int _0^1 {\mathbb {E}}_{\tau }\Big [\log p(D|{\varvec{\theta }},{\mathcal {M}})\Big ] d\tau \approx \int _0^1 \log p(D|{\varvec{\theta }}^{(\tau )},{\mathcal {M}}) d\tau \nonumber \\\approx & {} \sum _{k=2}^K \frac{\tau _k-\tau _{k-1}}{2} \Big \{ \log p(D|{\varvec{\theta }}^{(\tau _k)},{\mathcal {M}}) + \log p(D|{\varvec{\theta }}^{(\tau _{k-1})},{\mathcal {M}}) \Big \}\nonumber \\ \end{aligned}$$

where $\theta ^{(\tau )}$ is a single draw from the power posterior defined in Eq. (4), and $0=\tau _1<\tau _2<\cdots <\tau _K=1$. The computational resources gained are used to choose *K* orders of magnitude larger than in equilibrium TI,^3^ with the implication that $(\tau _k-\tau _{k-1}) \rightarrow 0$ and discretisation errors in numerical integration are avoided. This scheme was originally proposed in statistical physics (Schlitter and Husmeier 1992) under the name *non-equilibrium thermodynamic integration* (NETI), and is conceptionally similar to annealed importance sampling (Neal 2001). The underlying rationale is as follows: rather than use the computational resources for the computation of the expectation value at a limited number of discretisation points—and incur discretisation errors—spread the computational resources over the whole “temperature” range and use as fine a discretisation as possible. This avoids the problem that had to be addressed in Friel et al. (2014): how to select the inverse temperatures and minimise the numerical integration error in standard TI. The price to pay is a relaxation error as a consequence of the non-equilibrium nature of the method, as discussed by Schlitter and Husmeier (1992). The authors proposed a scheme for correcting this relaxation error, by running simulations over different simulation lengths $N_{iter}$, regressing the estimates against an approximate upper bound on the relaxation error ${\mathcal {R}}$, and then extrapolating for ${\mathcal {R}}\rightarrow 0$. In preliminary investigations omitted from the present article, we found that a single simulation with an increased value of $N_{iter}$ matching the total computational costs of the extrapolation scheme achieved similar results, and we used this conceptionally simpler approach in all our studies.^4^

### Novel thermodynamic integration for Bayes factors

When comparing two models, we are typically interested in the Bayes factor $p(D|{\mathcal {M}}_2)/p(D|{\mathcal {M}}_1)$. The standard approach is to apply thermodynamic integration to both models ${\mathcal {M}}_1$ and ${\mathcal {M}}_2$ separately, by independently inversely annealing the prior distributions to the respective posterior distributions. This approach ignores the fact that both models usually have many aspects in common and share certain parameters. This applies particularly to nested models, where all the parameters of the less complex model are also included in the more complex model. One would expect to reduce the estimation uncertainty by following a direct transition path from the posterior distribution of the less complex model to that of the more complex model, rather than transiting through the uninformative prior distribution twice. Consider two models ${\mathcal {M}}_1$ and ${\mathcal {M}}_2$ with joint parameter vector ${\varvec{\theta }}$ and a joint parameter prior $p({\varvec{\theta }}|{\mathcal {M}}_1,{\mathcal {M}}_2)$ defined such that it reduces to the parameter priors for the separate models by marginalisation:

12 $$\begin{aligned} p({\varvec{\theta }}|{\mathcal {M}}_1) = \int _{{\mathcal {M}}_2/{\mathcal {M}}_1} p({\varvec{\theta }}|{\mathcal {M}}_1,{\mathcal {M}}_2) d{\varvec{\theta }}, \quad p({\varvec{\theta }}|{\mathcal {M}}_2) = \int _{{\mathcal {M}}_1/{\mathcal {M}}_2} p({\varvec{\theta }}|{\mathcal {M}}_1,{\mathcal {M}}_2) d{\varvec{\theta }} \end{aligned}$$

where ${\mathcal {M}}_2/{\mathcal {M}}_1$ is the subset of parameters contained in model ${\mathcal {M}}_2$, but not in model ${\mathcal {M}}_1$, and ${\mathcal {M}}_1/{\mathcal {M}}_2$ is the subset of parameters contained in model ${\mathcal {M}}_1$, but not in model ${\mathcal {M}}_2$. A mathematically more accurate notation would split ${\varvec{\theta }}$ into three subsets, ${\varvec{\theta }}=\{{\varvec{\theta }}_1,{\varvec{\theta }}_2,{\varvec{\theta }}_{12}\}$ such that ${\varvec{\theta }}_1 \in {\mathcal {M}}_1/{\mathcal {M}}_2$, ${\varvec{\theta }}_2 \in {\mathcal {M}}_2/{\mathcal {M}}_1$ and ${\varvec{\theta }}_{12} \in {\mathcal {M}}_1\cap {\mathcal {M}}_2$. Eq. (12) implies that $p({\varvec{\theta }}|{\mathcal {M}}_1)=p({\varvec{\theta }}_1,{\varvec{\theta }}_{12}|{\mathcal {M}}_1)$ and $p({\varvec{\theta }}|{\mathcal {M}}_2)=p({\varvec{\theta }}_2,{\varvec{\theta }}_{12}|{\mathcal {M}}_2)$. For that reason we can use a mathematically redundant but less opaque notation that does not make the partition ${\varvec{\theta }}=\{{\varvec{\theta }}_1,{\varvec{\theta }}_2,{\varvec{\theta }}_{12}\}$ explicit. Define the tempered posterior distribution

13 $$\begin{aligned} p_{\tau }({\varvec{\theta }}|D,{\mathcal {M}}_1,{\mathcal {M}}_2) = \frac{p(D|{\varvec{\theta }},{\mathcal {M}}_2)^{\tau } p(D|{\varvec{\theta }},{\mathcal {M}}_1)^{1-\tau } p({\varvec{\theta }}|{\mathcal {M}}_1,{\mathcal {M}}_2)}{Z(D|\tau ,{\mathcal {M}}_1,{\mathcal {M}}_2)} \end{aligned}$$

where

14 $$\begin{aligned} Z(D|\tau ,{\mathcal {M}}_1,{\mathcal {M}}_2) = \int \left( \frac{p(D|{\varvec{\theta }},{\mathcal {M}}_2)}{p(D|{\varvec{\theta }},{\mathcal {M}}_1)} \right) ^{\tau } p(D|{\varvec{\theta }},{\mathcal {M}}_1) p({\varvec{\theta }}|{\mathcal {M}}_1,{\mathcal {M}}_2) d{\varvec{\theta }} \end{aligned}$$

From Eq. (12) we get:

15 $$\begin{aligned} p(D|{\mathcal {M}}_1) = Z(D|\tau =0,{\mathcal {M}}_1,{\mathcal {M}}_2), \quad p(D|{\mathcal {M}}_2) = Z(D|\tau =1,{\mathcal {M}}_1,{\mathcal {M}}_2) \end{aligned}$$

Taking the derivative of the partition function in Eq. (14) gives:

16 $$\begin{aligned}&\frac{d}{d\tau } \log Z(D|\tau ,{\mathcal {M}}_1,{\mathcal {M}}_2) = \frac{1}{Z(D|\tau ,{\mathcal {M}}_1,{\mathcal {M}}_2)} \frac{d}{d\tau } Z(D|\tau ,{\mathcal {M}}_1,{\mathcal {M}}_2) \nonumber \\&\quad = \frac{1}{Z(D|\tau ,{\mathcal {M}}_1,{\mathcal {M}}_2)} \int \frac{d}{d\tau } \left( \frac{p(D|{\varvec{\theta }},{\mathcal {M}}_2)}{p(D|{\varvec{\theta }},{\mathcal {M}}_1)} \right) ^{\tau } p(D|{\varvec{\theta }},{\mathcal {M}}_1)\nonumber \\&\qquad \times \, p({\varvec{\theta }}|{\mathcal {M}}_1,{\mathcal {M}}_2) d{\varvec{\theta }} \nonumber \\&\quad = \int \log \left( \frac{p(D|{\varvec{\theta }},{\mathcal {M}}_2)}{p(D|{\varvec{\theta }},{\mathcal {M}}_1)} \right) \frac{p(D|{\varvec{\theta }},{\mathcal {M}}_2)^{\tau } p(D|{\varvec{\theta }},{\mathcal {M}}_1)^{1-\tau } p({\varvec{\theta }}|{\mathcal {M}}_1,{\mathcal {M}}_2)}{Z(D|\tau ,{\mathcal {M}}_1,{\mathcal {M}}_2)} d{\varvec{\theta }} \nonumber \\&\quad = \int p_{\tau }({\varvec{\theta }}|D,{\mathcal {M}}_1,{\mathcal {M}}_2) \log \left( \frac{p(D|{\varvec{\theta }},{\mathcal {M}}_2)}{p(D|{\varvec{\theta }},{\mathcal {M}}_1)} \right) d{\varvec{\theta }} \nonumber \\&\quad = {\mathbb {E}}_{\tau }\left[ \log \left( \frac{p(D|{\varvec{\theta }},{\mathcal {M}}_2)}{p(D|{\varvec{\theta }},{\mathcal {M}}_1)}\right) \right] \end{aligned}$$

Combining Eqs. (15, 16) gives the following thermodynamic integral for the log Bayes factor:

17 $$\begin{aligned} \log \left( \frac{p(D|{\mathcal {M}}_2)}{p(D|{\mathcal {M}}_1)}\right)= & {} \log Z(D|\tau =1,{\mathcal {M}}_1,{\mathcal {M}}_2) - \log Z(D|\tau =0,{\mathcal {M}}_1,{\mathcal {M}}_2) \nonumber \\= & {} \int _0^1 \left[ \frac{d}{d\tau } \log Z(D|\tau ,{\mathcal {M}}_1,{\mathcal {M}}_2)\right] d \tau \nonumber \\= & {} \int _0^1 {\mathbb {E}}_{\tau }\left[ \log \left( \frac{p(D|{\varvec{\theta }},{\mathcal {M}}_2)}{p(D|{\varvec{\theta }},{\mathcal {M}}_1)}\right) \right] d \tau \end{aligned}$$

Again, we follow the idea of non-equilibrium thermodynamic integration and make the approximation

18 $$\begin{aligned} \log \left( \frac{p(D|{\mathcal {M}}_2)}{p(D|{\mathcal {M}}_1)}\right)\approx & {} \int _0^1 \left[ \log \left( \frac{p(D|{\varvec{\theta }}^{(\tau )},{\mathcal {M}}_2)}{p(D|{\varvec{\theta }}^{(\tau )},{\mathcal {M}}_1)}\right) \right] d \tau \nonumber \\\approx & {} \sum _{k=2}^K \frac{\tau _k-\tau _{k-1}}{2} \Bigg \{ \log \left( \frac{p(D|{\varvec{\theta }}^{(\tau _k)},{\mathcal {M}}_2)}{p(D|{\varvec{\theta }}^{(\tau _k)},{\mathcal {M}}_1)}\right) +\log \left( \frac{p(D|{\varvec{\theta }}^{(\tau _{k-1})},{\mathcal {M}}_2)}{p(D|{\varvec{\theta }}^{(\tau _{k-1})},{\mathcal {M}}_1)}\right) \Bigg \}\nonumber \\ \end{aligned}$$

where ${\varvec{\theta }}^{(\tau )}$ is a single draw from the tempered posterior distribution defined in Eq. (13), $0=\tau _1<\tau _2<\cdots <\tau _K=1$, $K\gg 1$, and $(\tau _{k}-\tau _{k-1})\ll 1$.

In comparison with statistical physics, the proposed scheme corresponds to the direct computation of a free energy difference (Schlitter 1991; Schlitter and Husmeier 1992), which is more efficient, in terms of reduced estimation variance for given computational costs, than computing the difference of two separately computed standard free energies. The analogy from classical statistics is model comparison via a paired test, which is known to have higher power than an unpaired test.

In what follows, we refer to the estimator defined by Eq. (18) as NETI-DIFF. We describe how to compute the variance of this estimator in the Appendix 7.2.

### Metropolis–Hastings scheme

The implementation of a Metropolis-Hastings scheme to target the distribution in (13) is straightforward. Given the current parameters ${\varvec{\theta }}$, sample new parameters ${{\tilde{{\varvec{{\theta }}}}}}$ from a proposal distribution $q({{\tilde{{\varvec{{\theta }}}}}}|{\varvec{\theta }})$, and accept these new parameters with the following acceptance probability:

19 $$\begin{aligned} a({{\tilde{{\varvec{{\theta }}}}}}|{\varvec{\theta }})= \min \left\{ \frac{p(D|{{\tilde{{\varvec{{\theta }}}}}},{\mathcal {M}}_2)^{\tau } p(D|{{\tilde{{\varvec{{\theta }}}}}},{\mathcal {M}}_1)^{1-\tau } p({{\tilde{{\varvec{{\theta }}}}}}|{\mathcal {M}}_1,{\mathcal {M}}_2)q({\varvec{\theta }}|{{\tilde{{\varvec{{\theta }}}}}})}{p(D|{\varvec{\theta }},{\mathcal {M}}_2)^{\tau } p(D|{\varvec{\theta }},{\mathcal {M}}_1)^{1-\tau } p({\varvec{\theta }}|{\mathcal {M}}_1,{\mathcal {M}}_2)q({{\tilde{{\varvec{{\theta }}}}}}|{\varvec{\theta }})} ,1 \right\} \end{aligned}$$

Otherwise, set ${{\tilde{{\varvec{{\theta }}}}}}={\varvec{\theta }}$, and follow this scheme iteratively.

### Gibbs sampling for linear models

Consider a standard linear model with parameter vector ${\varvec{\theta }}$, design matrix $\mathbf{{D}}$, and prior distribution

20 $$\begin{aligned} p({\varvec{\theta }}|\delta ^2,\sigma ^2) = N(\varvec{\mu }_0,\sigma ^2\delta ^2\mathbf{I}) \end{aligned}$$

The data, $D=\{y_1,\ldots ,y_{T}\}$ or $\mathbf{Y}= (y_1,\ldots ,y_{T})^{{}^{\mathrm{T}}}$, are assumed to be obtained under the assumption of independent and identically distributed normal noise, with variance $\sigma ^2$:

21 $$\begin{aligned} p(\mathbf{y}|{\varvec{\theta }},\sigma ^2)= N(\mathbf{{D}}{\varvec{\theta }},\sigma ^2\mathbf{I}) \end{aligned}$$

We want to compare two competing models ${\mathcal {M}}_1$ and ${\mathcal {M}}_2$, represented by two alternative design matrices $\mathbf{{D}}^{(1)}$ and $\mathbf{{D}}^{(2)}$:

22 $$\begin{aligned} p(\mathbf{y}|{\varvec{\theta }},\sigma ^2,{\mathcal {M}}_1)= N(\mathbf{{D}}^{(1)}{\varvec{\theta }},\sigma ^2\mathbf{I}), \quad p(\mathbf{y}|{\varvec{\theta }},\sigma ^2,{\mathcal {M}}_2)= N(\mathbf{{D}}^{(2)}{\varvec{\theta }},\sigma ^2\mathbf{I}) \end{aligned}$$

For notational compactness we choose a representation that leaves the dimension of ${\varvec{\theta }}$ invariant with respect to changing model dimensions by padding obsolete entries in the design matrix with zeros. For instance, to compare the models ${\mathcal {M}}_1{:} y= \theta _1 x_1 + \theta _2 x_2$, and ${\mathcal {M}}_2{:} y= \theta _1 x_1 + \theta _3 x_3 + \theta _4 x_4$ based on a data set of *n* observations $\{y_t,x_{1,t},x_{2,t},x_{3,t},x_{4,t}\}$, $t=1,\ldots ,n$, we get the following design matrices:

$$\begin{aligned} \mathbf{{D}}^{(1)}= \left( \begin{array}{cccc} x_{1,1} &{}\quad x_{2,1} &{}\quad 0 &{}\quad 0 \\ x_{1,2} &{}\quad x_{2,2} &{}\quad 0 &{}\quad 0 \\ \vdots &{}\quad \vdots &{}\quad \vdots &{}\quad \vdots \\ x_{1,n} &{}\quad x_{2,n} &{}\quad 0 &{}\quad 0 \\ \end{array} \right) , \; \mathbf{{D}}^{(2)}= \left( \begin{array}{cccc} x_{1,1} &{}\quad 0 &{}\quad x_{3,1} &{}\quad x_{4,1} \\ x_{1,2} &{}\quad 0 &{}\quad x_{3,2} &{}\quad x_{4,2} \\ \vdots &{}\quad \vdots &{}\quad \vdots &{}\quad \vdots \\ x_{1,n} &{}\quad 0 &{}\quad x_{3,n} &{}\quad x_{4,n} \\ \end{array} \right) \end{aligned}$$

From (13) we get

23 $$\begin{aligned} p_{\tau }({\varvec{\theta }}|D,{\mathcal {M}}_1,{\mathcal {M}}_2)\propto & {} p(D|{\varvec{\theta }},{\mathcal {M}}_2)^{\tau } p(D|{\varvec{\theta }},{\mathcal {M}}_1)^{1-\tau } p({\varvec{\theta }}|{\mathcal {M}}_1,{\mathcal {M}}_2)\nonumber \\\propto & {} N(\mathbf{{D}}^{(1)}{\varvec{\theta }},\sigma ^2\mathbf{I})^{1-\tau } N(\mathbf{{D}}^{(2)}{\varvec{\theta }},\sigma ^2\mathbf{I})^{\tau } N({\varvec{\mu }}_0,\sigma ^2\delta ^2\mathbf{I}) \nonumber \\\propto & {} \exp \left( \frac{-(1-\tau )}{2\sigma ^2} \left[ \mathbf{{D}}^{(1)}{\varvec{\theta }}-\mathbf{y}\right] ^{{}^{\top }} \left[ \mathbf{{D}}^{(1)}{\varvec{\theta }}-\mathbf{y}\right] \right) \nonumber \\&\quad \exp \left( \frac{-\tau }{2\sigma ^2} \left[ \mathbf{{D}}^{(2)}{\varvec{\theta }}-\mathbf{y}\right] ^{{}^{\top }} \left[ \mathbf{{D}}^{(2)}{\varvec{\theta }}-\mathbf{y}\right] \right) \end{aligned}$$

24 $$\begin{aligned}&\exp \left( \frac{-1}{2\sigma ^2\delta ^2} [{\varvec{\theta }}-\varvec{\mu }_0]^{{}^{\top }} [{\varvec{\theta }}-\varvec{\mu }_0] \right) \nonumber \\= & {} \exp \left( \frac{-1}{2\sigma ^2} {\varvec{\theta }}^{{}^{\top }} \left[ \tau \{\mathbf{{D}}^{(2)}\}^{{}^{\top }}\mathbf{{D}}^{(2)}+ (1-\tau )\{\mathbf{{D}}^{(1)}\}^{{}^{\top }}\mathbf{{D}}^{(1)}+ \delta ^{-2}{} \mathbf{I}\right] {\varvec{\theta }}\right) \nonumber \\&\exp \left( \frac{1}{\sigma ^2} {\varvec{\theta }}^{{}^{\top }} \left( \left[ \tau \{\mathbf{{D}}^{(2)}\}^{{}^{\top }} + (1-\tau )\{\mathbf{{D}}^{(1)}\}^{{}^{\top }} \right] \mathbf{y}+ \delta ^{-2}\varvec{\mu }_{0}\right) \right) C(\mathbf{y})\nonumber \\ \end{aligned}$$

where the factor $C(\mathbf{y})$ does not depend on ${\varvec{\theta }}$. Comparing this with the identity

$$\begin{aligned} N({\varvec{\theta }}|\varvec{\mu },\sigma ^2\mathbf{H}^{-1})\propto & {} \exp \left( \frac{-1}{2\sigma ^2} [{\varvec{\theta }}-\varvec{\mu }]^{{}^{\top }}{} \mathbf{H} [{\varvec{\theta }}-\varvec{\mu }] \right) \nonumber \\= & {} \exp \left( \frac{-1}{2\sigma ^2} {\varvec{\theta }}^{{}^{\top }}{} \mathbf{H}{\varvec{\theta }}\right) \exp \left( \frac{1}{\sigma ^2} {\varvec{\theta }}^{{}^{\top }}{} \mathbf{H}\varvec{\mu }\right) C(\varvec{\mu }) \end{aligned}$$

we get:

25 $$\begin{aligned} p_{\tau }({\varvec{\theta }}|D,{\mathcal {M}}_1,{\mathcal {M}}_2) \; = \; N({\varvec{\theta }}|\varvec{\mu },\sigma ^2\mathbf{H}^{-1}) \end{aligned}$$

where

26 $$\begin{aligned} \mathbf{H}= & {} \tau \{\mathbf{{D}}^{(2)}\}^{{}^{\top }} \mathbf{{D}}^{(2)}+ (1-\tau )\{\mathbf{{D}}^{(1)}\}^{{}^{\top }} \mathbf{{D}}^{(1)}+ \delta ^{-2} \mathbf{I}, \nonumber \\ \varvec{\mu }= & {} \mathbf{H}^{-1} \left( \left[ \tau \{\mathbf{{D}}^{(2)}\}^{{}^{\top }} + (1-\tau )\{\mathbf{{D}}^{(1)}\}^{{}^{\top }} \right] \mathbf{y}+ \delta ^{-2} {\varvec{\mu }}_0 \right) \end{aligned}$$

Hence, we can directly sample ${\varvec{\theta }}$ from the tempered conditional distributions in a Gibbs sampling scheme without having to resort to Metropolis-Hastings. For linear models where the variance $\sigma ^2$ is not known and has to be sampled from the tempered posterior distribution too, we refer to Appendix 7.6.

### Sigmoid inverse temperature ladder

Given a single model ${\mathcal {M}}$, conventional TI follows an inverse annealing path from the prior $p({\varvec{\theta }}|{\mathcal {M}})$ to the posterior $p({\varvec{\theta }}|{\mathcal {M}},D)$, symbolically $p({\varvec{\theta }}|{\mathcal {M}})\rightarrow p({\varvec{\theta }}|{\mathcal {M}},D)$. Unlike TI, NETI-DIFF is based on a direct transition from the posterior of one model ${\mathcal {M}}_1$ to the posterior of another model ${\mathcal {M}}_2$, $p({\varvec{\theta }}|{\mathcal {M}}_1,D)\rightarrow p({\varvec{\theta }}|{\mathcal {M}}_2,D)$. For nested models, e.g. ${\mathcal {M}}_1 \subset {\mathcal {M}}_2$, we start at the less complex model ${\mathcal {M}}_1$ and move towards the more complex model ${\mathcal {M}}_2$, e.g. using the power-law inverse temperature ladder, defined in Eq. (8). For a power $\alpha >1$ the distances $\tau _{i+1}-\tau _{i}$ between neighbouring discretisation points $\tau _{k}$ and $\tau _{k+1}$ increase in *k* and the discretisation points will be concentrated around the nested model, ${\mathcal {M}}_1$ ($\tau =0$), and fewer points will be set near ${\mathcal {M}}_2$ ($\tau =1$). However, in many applications non-nested models have to be compared, and it is then not clear which of the two models should be used as starting point. Imbalances can be avoided by choosing a sigmoid inverse temperature ladder, such that the discretisation points are mirrored at the midpoint $\tau ^{\star }=0.5$ of the interval [0, 1]. Every discretisation point $\tau <0.5$ closer to ${\mathcal {M}}_1$ then has its counterpart $\tau ^{\star }=1-\tau$ with the same distance $\tau$ to ${\mathcal {M}}_2$, and vice-versa.

To obtain a sigmoid inverse temperature ladder for NETI-DIFF we apply the following procedure. We first specify 50% of the discretisation points $\tau _1<\cdots <\tau _{\frac{N_{iter}}{2}}$ within the interval [0, 0.5], and then we mirror the ladder at the midpoint $\tau =0.5$.^5^ This yields the remaining $50\%$ of the discretisation points, $\tau _{\frac{N_{iter}}{2}+i}=1-\tau _{\frac{N_{iter}}{2}+1-i}$ ($i=1,\ldots ,\frac{N_{iter}}{2}$). As we want the first $50\%$ of the discretisation points to get as close as possible to the midpoint $\tau =0.5$ subject to a power law with power $\alpha$, we determine the minimal integer $N^{\star }$ such that

27 $$\begin{aligned} \tau _i := \left( \frac{i}{N^{\star }}\right) ^\alpha < 0.5 \quad (i=1,\ldots ,\frac{N_{iter}}{2}) \end{aligned}$$

The solution is: $N^{\star } = \lfloor x^{\star } \rfloor$, where

28 $$\begin{aligned} \left( \frac{N_{iter}}{2x^{\star }}\right) ^\alpha = 0.5 \Leftrightarrow x^{\star } = \frac{N_{iter}}{2} \cdot 0.5^{-\frac{1}{\alpha }} \end{aligned}$$

## Benchmark problems and data

We have evaluated the proposed method on four benchmark data sets. Given data $D$ the goal is to estimate the log Bayes factor ${\mathcal {B}}$ between two models ${\mathcal {M}}_1$ and ${\mathcal {M}}_2$. We assume the models to be equally likely a priori, $p({\mathcal {M}}_1)=p({\mathcal {M}}_2)$, so that the Bayes factor is the ratio of marginal likelihoods:

29 $$\begin{aligned} {\mathcal {B}}({\mathcal {M}}_1,{\mathcal {M}}_2) = \log \left\{ \frac{ p({\mathcal {M}}_2|D)}{p({\mathcal {M}}_1|D)} \right\} = \log \left\{ \frac{ p(D|{\mathcal {M}}_2)}{p(D|{\mathcal {M}}_1)} \right\} \end{aligned}$$

For nonuniform prior distributions, $p({\mathcal {M}}_1)\ne p({\mathcal {M}}_2)$, it is straightforward to add the correction factor $\log p({\mathcal {M}}_2)/p({\mathcal {M}}_1)$, which is computationally cheap compared to the marginal likelihood ratio.

For method evaluation, we need to compare with a ground truth. For a linear model, we have a proper ground truth, as the Bayes factor can be computed analytically. This applies to the Radiata pine data (Sect. 4.1) and the Radiocarbon data (Sect. 4.3). For the Pima Indian data (Sect. 4.2), we use a generalised linear model, and for the biopathway data (Sect. 4.4), we use a nonlinear model. In these cases, a closed-form solution of the Bayes factor does not exist. For the Pima Indian data, we follow the method suggested in Friel et al. (2014) and use the numerical result from a very long MCMC run as an approximate gold standard. For the biopathway data, we use the knowledge of the true interaction structure of the system as a surrogate gold standard and assess the performance in terms of network reconstruction accuracy. We think this provides an adequate balance between using linear models, for which a strong ground truth exists, and generalised linear/non-linear models, for which a strong ground truth is intrinsically unavailable, and a weaker surrogate ground truth has to be used instead.

### Radiata pine

The Radiata pine data have been used in Friel et al. (2014) and were originally published in Williams (1959). Like Friel et al. (2014) we focus on the log Bayes factor between two competing non-nested linear regression models for explaining the ’maximum compression strength’ *y* of $n=42$ Radiata pine specimens. Both linear models contain an intercept and one single covariate. The first model (${\mathcal {M}}_1$) uses the ’density’ $x_1$ and the second model (${\mathcal {M}}_2$) the ’adjusted density’ $x_2$ of the specimen. After standardizing the observation vectors ${\mathbf{x}}_1$ and ${\mathbf{x}}_2$ of the two covariates to mean 0, the likelihood of model ${\mathcal {M}}_k$ ($k=1,2$) is:

30 $$\begin{aligned} {\mathcal {M}}_k: p({\mathbf{y}}|{\varvec{\theta }}^{(k)},\sigma ^2) = N({\mathbf{D}}^{(k)} {\varvec{\theta }}^{(k)}, \sigma ^2 {\mathbf{I}}) \end{aligned}$$

where ${\mathbf{y}}$ is the vector of the observed ’maximum compression strengths’, ${\mathbf{D}}^{(k)}=({\mathbf{1}},{\mathbf{x}}_k)$ is the *n*-by-2 design matrix and ${\varvec{\theta }}^{(k)}$ is the 2-dimensional vector of regression coefficients of model ${\mathcal {M}}_k$. Both models share the intercept parameter $\theta _0$, but differ w.r.t. the second parameter, i.e. ${\varvec{\theta }}^{(k)}=(\theta _0,\theta _k)^{\top }$. For comparability we use exactly the same Bayesian model as in Friel et al. (2014), where an inverse Gamma prior is imposed on the noise variance: $p(\sigma ^{-2})= GAM(3,2\cdot 300^2 )$ and Gaussian priors are used for the regression coefficient vectors:^6^

31 $$\begin{aligned} {\mathcal {M}}_k: p({\varvec{\theta }}^{(k)}) =N \left( \left( \begin{array}{r}3000\\ 185\\ \end{array} \right) , \left( \begin{array}{cc}0.06^{-1}&{}0\\ 0&{}6^{-1}\end{array} \right) \right) \end{aligned}$$

This is a model with fully conjugate priors, so that the marginal likelihoods $p({\mathbf{y}}|{\mathcal {M}}_k)$ can be computed in closed form (Friel et al. 2014). With Eq. (29) we obtain for the log Bayes factor ${\mathcal {B}}({\mathcal {M}}_1,{\mathcal {M}}_2)=8.8571$. Like Friel et al. (2014) we apply Gibbs sampling and re-sample the model parameters iteratively from their full conditional distributions: $p(\sigma ^2|{\mathbf{y}},{\varvec{\theta }}^{(k)})$ and $p({\varvec{\theta }}^{(k)}|{\mathbf{y}},\sigma ^2)$.

### Pima Indians

The Pima Indians data have also been used in Friel et al. (2014) and were originally published in Smith et al. (1988). Like Friel et al. (2014) we focus on the log Bayes factor between two nested logistic regression models for explaining the binary ’diabetes disease status’ *y* of $n=532$ female Pima Indians. The first model (${\mathcal {M}}_1$) contains an intercept and 4 covariates, namely ’the number of pregnancies’, ’the plasma glucose concentration’, ’the body mass index’, and ’the diabetes pedigree function’, while the second model (${\mathcal {M}}_2$) extends model ${\mathcal {M}}_1$ by including one additional covariable ’age’. After standardizing all covariates to mean 0 and variance 1, the likelihood of model ${\mathcal {M}}_k$ ($k=1,2$) is:

$$\begin{aligned} {\mathcal {M}}_k: p({\mathbf{y}}| {\varvec{\theta }}^{(k)} ) = \prod _{i=1}^{n} \frac{\left\{ \exp (-{\mathbf{x}}_{i,k}^{\top }{\varvec{\theta }}^{(k)}) \right\} ^{y_i} }{1+\exp (-{\mathbf{x}}_{i,k}^{\top }{\varvec{\theta }}^{(k)})} \end{aligned}$$

where the *i*-th element of ${\mathbf{y}}$, $y_i\in \{0,1\}$, is the diabetes status of female *i*, ${\mathbf{x}}_{i,k}$ is the corresponding vector of covariates, including an initial 1 for the intercept, and ${\varvec{\theta }}^{(k)}$ is the vector of regression coefficients of dimension $m=5$ (${\mathcal {M}}_1$) or $m=6$ (${\mathcal {M}}_2$). Again we follow Friel et al. (2014) and impose the following Gaussian priors on the regression coefficient vectors: $p({\varvec{\theta }}^{(k)}|\delta ^2) = N({\mathbf{0}}, \delta ^2 {\mathbf{I}} )$, where $\delta ^2=100$ gives rather uninformative priors. For the logistic regression neither the marginal likelihoods nor the full conditional distributions can be computed in closed form. We therefore use the Metropolis Hastings based Markov chain Monte Carlo (MCMC) sampling scheme from Friel et al. (2014), which employs the following proposal mechanism: In each iteration a new candidate regression coefficient vector is obtained by adding a sample ${\mathbf{u}}$ from an *m*-dimensional multivariate Gaussian distribution to the current vector ${\varvec{\theta }}^{(k)}$. The Gaussian distribution of ${\mathbf{u}}$ has a zero mean vector and a diagonal covariance matrix, whose diagonal entries $d_1,\ldots ,d_m$ depend on the inverse temperature $\tau \in [0,1]$ of the power posterior. For the TI approaches we set: $d_i=\min \{0.01\tau ^{-1},100\}$, as in Friel et al. (2014). For the proposed NETI-DIFF approach we use $d_6=\min \{0.01 \tau ^{-1},100\}$, while we fix the first five diagonal entries $d_1,\ldots ,d_5=0.01$. This modification is required, as the first five regression coefficients appear in both models ${\mathcal {M}}_1$ and ${\mathcal {M}}_2$. That is, they effectively appear constantly with inverse temperature $\tau =1$ throughout NETI-DIFF simulations. The marginal likelihoods cannot be computed in closed-form. We therefore use those values reported in Friel et al. (2014), which were obtained from long TI simulations, as gold-standard: $\log \{p({\mathbf{y}}|{\mathcal {M}}_1)\}=-257.2342$ and $\log \{p({\mathbf{y}}|{\mathcal {M}}_2)\}=-259.8519$. Equation (29) yields the log Bayes factor: ${\mathcal {B}}({\mathcal {M}}_1,{\mathcal {M}}_2)=-2.6177$.

### Radiocarbon dating

We use the Radiocarbon data from Pearson and Qua (1993) to compute the Bayes factors among 10 nested linear regression models. For predicting the ’true calendar age’ *y* of $n=343$ Irish oaks from one single covariable: ’the Radiocarbon dating process’ *x*, we fit polynomial calibration curves ${\mathcal {M}}_i$ ($i=1,\ldots ,10$) of the following type:

32 $$\begin{aligned} {\mathcal {M}}_i: y = \theta _0 + \sum _{j=1}^i \theta _j x^j \end{aligned}$$

The likelihood of model ${\mathcal {M}}_i$ is then

33 $$\begin{aligned} {\mathcal {M}}_i: p({\mathbf{y}}|\sigma ^2,{\varvec{\theta }}^{(i)}) = N({\mathbf{D}}^{(i)} {\varvec{\theta }}^{(i)} , \sigma ^2 {\mathbf{I}}) \end{aligned}$$

where ${\mathbf{y}}$ is the vector of calendar ages, ${\varvec{\theta }}^{(i)}=(\theta _0,\theta _1,\ldots ,\theta _i)^{\top }$ is the vector of regression coefficients, and ${\mathbf{D}}^{(i)}$ is the *n*-by-$(i+1)$ design matrix. The first column of the design matrix consists entirely of ones (for the intercept), and the subsequent columns are built from the observation vector ${\mathbf{x}}$, ${\mathbf{D}}^{(i)}=({\mathbf{1}},{\mathbf{x}}^1,\ldots ,{\mathbf{x}}^i)$, where ${\mathbf{x}}^j$ denotes an element-wise power operation on ${\mathbf{x}}$. We impose conjugate priors on the parameters. For $\sigma ^2$ we use an inverse Gamma distribution: $p(\sigma ^{-2})= GAM(\frac{a}{2},\frac{b}{2})$, and on ${\varvec{\theta }}^{(i)}$ we impose Gaussian priors:

34 $$\begin{aligned} p({\varvec{\theta }}^{(i)}|\sigma ^2,\delta ^2) = N({\mathbf{0}},\sigma ^2\delta ^2 {\mathbf{I}}) \end{aligned}$$

For fixed hyperparameters *a*, *b*, and $\delta ^2$ the marginal likelihood for a model ${\mathcal {M}}$ with design matrix ${\mathbf{D}}$ is given by:

$$\begin{aligned} p({\mathbf{y}}|{\mathcal {M}}) = \frac{ \varGamma (\frac{n+a}{2}) \cdot b^{\frac{a}{2}} \cdot (b + {\mathbf{y}}^{\top } ({\mathbf{I}}+\delta ^2 {\mathbf{D}} {\mathbf{D}}^{\top })^{-1} {\mathbf{y}})^{-\frac{n+a}{2} } }{ \varGamma (\frac{a}{2}) \cdot \pi ^{\frac{n}{2}} \cdot \det \left( {\mathbf{I}} + \delta ^2 {\mathbf{D}} {\mathbf{D}}^{\top } \right) } \end{aligned}$$

so that the log Bayes factors ${\mathcal {B}}({\mathcal {M}}_{i},{\mathcal {M}}_{l})$ for two models ${\mathcal {M}}_i$ and ${\mathcal {M}}_l$ can be computed in closed form with Eq. (29). For the Radiocarbon data we fix $a=b=0.2$, $\delta ^2=1$, and we sample the parameters iteratively from their conditional distributions $p(\sigma ^2|{\mathbf{y}},{\varvec{\theta }}^{(i)})$ and $p({\varvec{\theta }}^{(i)}|{\mathbf{y}},\sigma ^2)$ with Gibbs sampling.

**Fig. 1:**
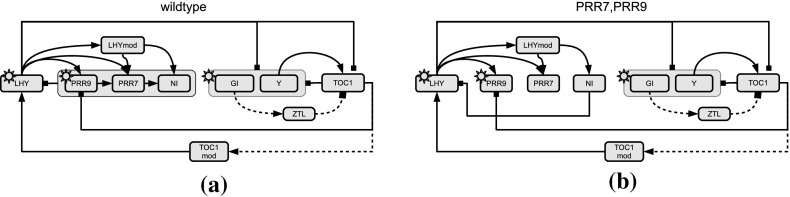
Gene regulatory networks of the circadian clock in *Arabidopsis thaliana*: wildtype and mutant. The network displayed in panel **a** is the P2010 network proposed by Pokhilko et al. (2010). Panel **b** shows a mutant network, in which the proteins PRR9 and PRR7 are dysfunctional and can no longer form a protein complex with NI. The *nodes* in the network represent proteins and genes, the *edges* indicate interactions. *Arrows* symbolize activations and *bars* inhibitions. *Solid lines* show protein-gene interactions; *dashed lines* show protein interactions. The regulatory influence of light is symbolized by a sun symbol. *Grey boxes* group sets of regulators or regulated components. Figure reproduced from Aderhold et al. (2014)

### Biopathway

The objective of the last application is model selection with respect to two alternative candidate interaction structures of ten genes in the circadian gene regulatory network of *Arabidopsis thaliana*, shown in Fig. 1. The statistical model used for inference is a semi-mechanistic Bayesian hierarchical model for transcriptional regulation (Aderhold et al. (2017)). Let $x_i(t)$ denote the mRNA concentration of gene *i* at time *t*, and $\pi _i$ the set of its regulators. For instance, in the gene network of Fig. 1a, the regulators of gene *PRR9* are two other genes, *TOC1* and *LHY*. So if $i=PRR9$, then $\pi _i=\{TOC1,LHY\}$. A regulator can either act as activator or as repressor, and we represent that with the binary variable $I_{u,i}$, with $I_{u,i}=1$ indicating that gene *u* is an activator for gene *i*, and $I_{u,i}=0$ indicating that gene *u* is an inhibitor for gene *i*. For the example above, *LHY* is an activator for *PRR9*, hence $I_{u,i}=1$, while *TOC1* is an inhibitor for *PRR9*, hence $I_{u,i}=0$. From the fundamental equation of transcriptional regulation based on Michaelis–Menten kinetics we have for the gradient of $x_i$ (Barenco et al. 2006):

35 $$\begin{aligned} \frac{d x_i(t)}{dt}|_{t=t^{\star }} = -v_{0,i} x_i(t^{\star }) + \sum _{u\in \pi _i} v_{u,i} \frac{I_{u,i} x_u(t^{\star })+(1-I_{u,i}) k_{u,i}}{x_u(t^{\star })+k_{u,i}} \end{aligned}$$

where the sum is over all genes *u* that are in the regulator set of $\pi _i$ of gene *i*. The first term, $-v_{0,i} x_i(t^{\star })$, takes the degradation of $x_i$ into account, while $v_{u,i}$ and $k_{u,i}$ are the *maximum reaction rate* and *Michaelis–Menten* parameters for the regulatory effect of gene $u\in \pi _i$ on gene *i*, respectively. See the supplementary material of Pokhilko et al. (2010, 2012) for similar examples in the mathematical biology literature. Without loss of generality, we now assume that $\pi _i$ is given by $\pi _i= \{x_1,\ldots ,x_s\}$. Equation (35) can then be written in vector notation:

36 $$\begin{aligned} \frac{d x_i(t)}{dt}|_{t=t^{\star }} = {\mathbf{D}}_{i,t^{\star }}^{\top } {\mathbf{V}}_i \end{aligned}$$

where ${\mathbf{V}}_i=(v_{0,i},v_{1,i}\ldots ,v_{s,i})^{\top }$ is the vector of the maximum reaction rate parameters, and the vector ${\mathbf{D}}_{i,t^{\star }}$ depends on the measured concentrations $x_u(t^{\star })$ and the Michaelis–Menten parameters $k_{u,i}$ ($u\in \pi _i$) via Eq. (35):

37 $$\begin{aligned} {\mathbf{D}}_{i,t^{\star }}^{\top } = \Big (-x_i(t^{\star }),\frac{I_{1,i} x_1(t^{\star })+(1-I_{1,i}) k_{1,i}}{x_1(t^{\star })+k_{1,i}},\ldots , \frac{I_{s,i} x_{s}(t^{\star })+(1-I_{s,i}) k_{s,i}}{x_s(t^{\star })+k_{s,i}} \Big )\nonumber \\ \end{aligned}$$

**Fig. 2:**
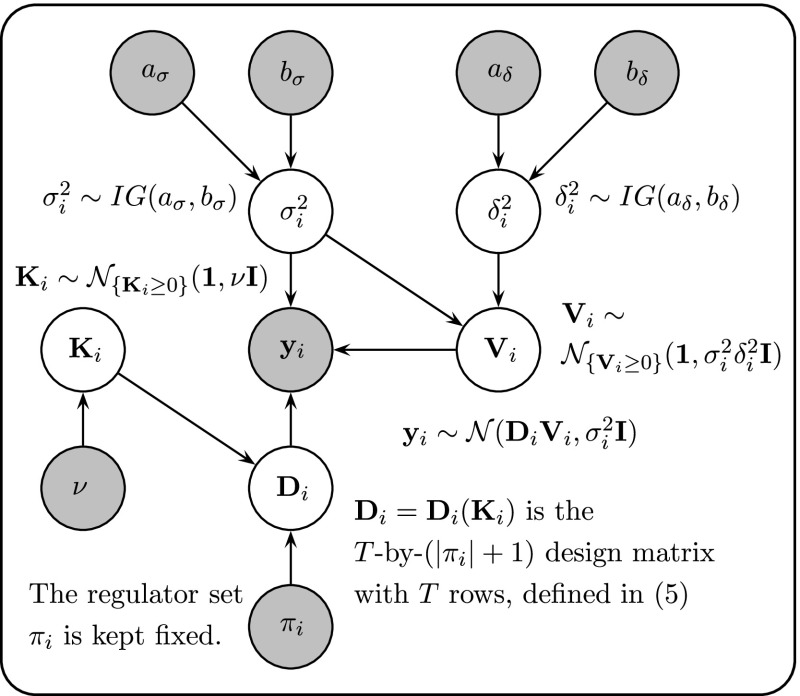
Hierarchical Bayesian model used for gene regulatory network reconstruction. *Grey nodes* refer to fixed quantities such as the observed response data or low-level hyperparameters. *White nodes* refer to quantities that can change, which includes the model parameters and high-level hyperparameters. Note that the design matrix $\mathbf{D}_i$ is not fixed because it depends on the Michaelis–Menten parameter vector $\mathbf{K}_i$

We combine the *s* Michaelis–Menten parameters $k_{u,i}$ in a vector ${\mathbf{K}}_i =(k_{1,i}\ldots ,k_{s,i})^{\top }$. For *n* time points $t^{\star }\in \{t_1,\ldots ,t_n\}$ we obtain *n* row vectors from Eq. (37), and we can arrange them successively in an *n*-by-$(|\pi _i|+1)$ design matrix ${\mathbf{D}}_i={\mathbf{D}}_i({\mathbf{K}}_i)$. The corresponding gradient vector is given by ${\mathbf{y}}_i:=(y_{i,1},\ldots ,y_{i,n})^{\top }$, where $y_{i,j}$ is the gradient of $x_i$ at time point $t_j$. With ${\mathbf{y}}_i$ being the response vector the likelihood is:

$$\begin{aligned} p({\mathbf{y}}_i|{\mathbf{K}}_i,{\mathbf{V}}_i,\sigma _i^2)= (2\pi \sigma _i^2)^{-\frac{n}{2}} e^{-\frac{1}{2\sigma ^2_{i}} ({\mathbf{y}}_i - {\mathbf{D}}_i {\mathbf{V}}_i)^{\top } ({\mathbf{y}}_i - {\mathbf{D}}_i {\mathbf{V}}_i)} \end{aligned}$$

where ${\mathbf{D}}_i={\mathbf{D}}_i({\mathbf{K}}_i)$ is the design matrix, given the Michaelis–Menten parameter vector ${\mathbf{K}}_i$. To ensure non-negative Michaelis–Menten parameters, truncated Normal prior distributions are used:

38 $$\begin{aligned} {\mathbf{K}}_i \sim {\mathcal {N}}_{\left\{ {\mathbf{K}}_i\ge 0 \right\} }({\mathbf{1}},\nu {\mathbf{I}}) \end{aligned}$$

where $\nu >0$ is a hyperparameter, and the subscript, $\left\{ {\mathbf{K}}_i\ge 0 \right\}$, indicates the truncation condition, i.e. that each element of ${\mathbf{K}}_i$ has to be non-negative. For the maximum reaction rates, we use a truncated ridge regression prior:

39 $$\begin{aligned} {\mathbf{V}}_i|\sigma ^2_i,\delta ^2_i \sim {\mathcal {N}}_{\left\{ {\mathbf{V}}_i\ge 0\right\} }( {\mathbf{1}}, \delta ^2_i \sigma _i^2 {\mathbf{I}} ) \end{aligned}$$

where $\delta ^2_i$ is a hyperparameter that regulates the prior strength. For $\sigma _i^2$ and $\delta ^2_i$ we use inverse Gamma priors, $\sigma _i^{2}\sim IG(a_{\sigma },b_{\sigma })$ and $\delta ^2_i \sim IG(a_{\delta },b_{\delta })$. A graphical model representation can be found in Fig. 2.

The posterior distribution of the parameters and hyperparameters has no closed-form solution, and we therefore resort to an MCMC scheme to sample from it. From the graphical model in Fig. 2 it can be seen that with the sole exception of the Michaelis–Menten parameters $\mathbf{K}_i$, the conditional distribution of each parameter conditional on its Markov blanket^7^ is of standard form (due to conjugacy) and can be sampled from directly. The MCMC scheme is therefore of the form of a Gibbs sampler, in which all parameters are sampled directly from their conditional distributions, except for $\mathbf{K}_i$, which is sampled via a Metropolis-Hastings within Gibbs step. The conditional distribution of the maximum rate parameter vector $\mathbf{V}_i$ is obtained from Eqs. (25–26) by replacing ${\varvec{\theta }}$ by $\mathbf{V}_i,$ and adding an index *i*, for association with gene *i*, to all other quantities except for the identity matrix $\mathbf{I}$ and the inverse temperature $\tau$. The derivation of the other conditional distributions is straightforward. Pseudo code of the standard MCMC algorithm can be found in Aderhold et al. (2017). Pseudo code of the modified MCMC algorithm integrated into the proposed NETI-DIFF scheme is provided in the Appendix, Table 2.

The data used for inference were obtained from Aderhold et al. (2014). These are synthetic gene expression time series, which were generated from a biologically realistic simulation of the molecular interactions in these networks, using the mathematical framework described in Guerriero et al. (2012) and implemented in the Biopepa software package (Ciocchetta and Hillston 2009). These time series correspond to gene expression measurements in 2h intervals over 24 h, repeated 11 times for different experimental conditions related to various gene knockouts. We repeated the simulations twice, for both of the two networks shown in Fig. 1. Hence, the true interaction network is known, which can be used to evaluate the accuracy of Bayesian model selection based on the modelling framework described above.

## Results

In this section, we compare the efficiency and accuracy of three algorithms: standard thermodynamic integration (TI-standard) and optimal thermodynamic integration (TI-optimal) for computing the log marginal likelihood, and the proposed non-equilibrium thermodynamic integration for directly targeting the difference of the log marginal likelihood (NETI-DIFF).

In TI-standard we compute, based on Eq. (5), the expectation of the log likelihood w.r.t. the power posterior, ${\mathbb {E}}_{\tau }[ \log p(D|{\varvec{\theta }},{\mathcal {M}})]$, for a set of a priori fixed inverse temperatures $\{\tau _i\}$, $i=1,\ldots ,K$, spaced according to the power law of Eq. (8). Following Friel et al. (2014) we have set $K \in \{10,20,50,100\}$ and $\alpha =5$ in Eq. (8). The log marginal likelihood is computed with the trapezoid rule (Eq. 7).

TI-optimal uses the two improvements proposed in Friel et al. (2014): the log marginal likelihood is computed with the improved numerical integration (Eq. 10), and the inverse temperatures are set iteratively according to an optimality criterion that aims to minimise the expected uncertainty; see Friel et al. (2014) for details.^8^

Finally, NETI-DIFF is the algorithm proposed in the present article.

For each inverse temperature $\tau$ in TI-standard and TI-optimal, we discarded the first 20% of the MCMC steps as burn-in (following Friel et al. (2014)). For NETI-DIFF, we discarded the first 1000 MCMC steps with the inverse temperature kept fixed at $\tau =0$, as burn-in.^9^ We recorded the total number of non-burn-in MCMC steps for all three algorithms, $N_{iter}$. As discussed in Appendix 7.7 this is a measure of the total computational complexity.

We repeated the MCMC simulations $N_{simu}=5$ times from different initialisations. Let ${\mathcal {B}}_i$ denote the log Bayes factor obtained from the *i*th MCMC simulation, and ${\mathcal {B}}_{true}$ the ‘true’ log Bayes factor. For the Bayesian linear regression models applied to the Radiata and Radiocarbon data, a closed-form expression for ${\mathcal {B}}_{true}$ is available. For the Bayesian logistic regression model applied to the Pima Indians data, and the hierarchical Bayesian model from Fig. 2 for biopathway data, the log Bayes factor is not analytically tractable, and ${\mathcal {B}}_{true}$ was obtained from a very long simulation, as in Friel et al. (2014). We assessed the intrinsic estimation *uncertainty* in terms of the variance:

40 $$\begin{aligned} {\mathbb {V}}= \frac{1}{N_{simu}-1} \sum _{i=1}^{N_{simu}} [{\mathcal {B}}_i - \overline{{\mathcal {B}}}]^2, \quad \quad \overline{{\mathcal {B}}} = \frac{1}{N_{simu}} \sum _{i=1}^{N_{simu}} {\mathcal {B}}_i \end{aligned}$$

and the *accuracy* in terms of the mean absolute error:

41 $$\begin{aligned} {\mathbb {A}}= \frac{1}{N_{simu}} \sum _{i=1}^{N_{simu}} |{\mathcal {B}}_i - {\mathcal {B}}_{true}| \end{aligned}$$

### Radiata pine and Pima Indians

We start our empirical evaluation study with the analysis of the Radiate pine data (Sect. 4.1) and the Pima Indians data (Sect. 4.2). These two data sets have been used in the literature before for the evaluation of the TI method proposed by Friel et al. (2014), and in both cases the goal is to estimate the Bayes factor between two competing Bayesian regression models. For the Radiata pine data we compare two non-nested linear regression models. For the Pima Indians data we compare two logistic regression models, where the first model, ${\mathcal {M}}_1$, is nested in the second, ${\mathcal {M}}_2$ . We apply the NETI-DIFF approach with a sigmoid inverse temperature ladder, defined in Sect. 3.6, and we instantiate NETI-DIFF such that in both applications the transition path runs from the first model, ${\mathcal {M}}_1$ ($\tau =0$), to the second, ${\mathcal {M}}_2$ ($\tau =1$). For the Pima Indians data this is the natural path, as ${\mathcal {M}}_1$ is nested within ${\mathcal {M}}_2$.

**Fig. 3:**
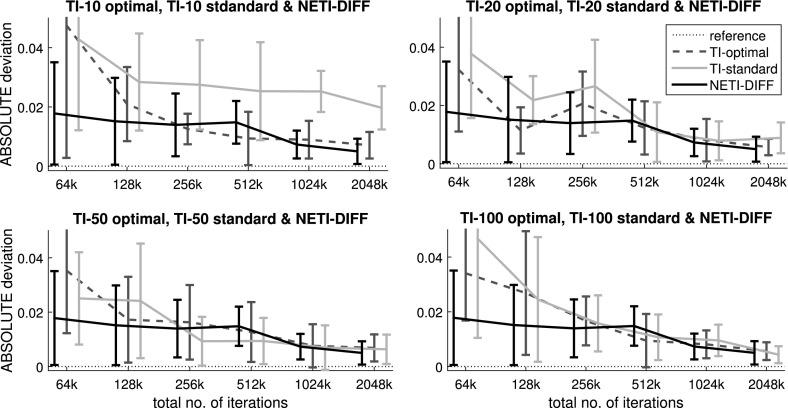
Average absolute error on the Radiata pine data: TI versus NETI-DIFF. The figure shows the average absolute deviation between the estimated and the true log Bayes factor in dependence on the total number of MCMC iterations $N_{iter}$. In each panel the same NETI-DIFF results are shown, while the two TI approaches (TI-standard and TI-optimal) were applied with different numbers of discretisation points (10, 20, 50 and 100). The *error bars* represent standard deviations. The *horizontal axes* give the total number of (power posterior) MCMC iterations, $N_{iter}$

**Fig. 4:**
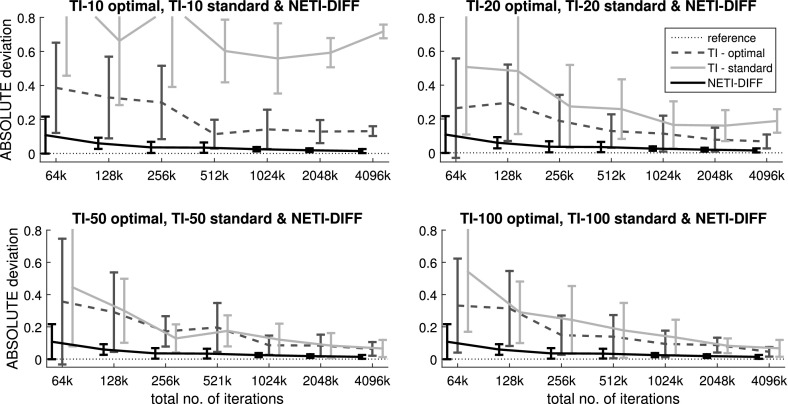
Average absolute error on the Pima Indians data: TI versus NETI-DIFF. The figure shows the average absolute deviation between the estimated and the true log Bayes factor in dependence on the total number of MCMC iterations $N_{iter}$. In each panel the same NETI-DIFF results are shown, while the two TI approaches (TI-standard and TI-optimal) were applied with different numbers of discretisation points (10, 20, 50 and 100). The *error bars* represent standard deviations. The *horizontal axes* give the total number of (power posterior) MCMC iterations, $N_{iter}$

Figures 3 and 4 show the average absolute deviations (Eq. 41) between the analytically computed log Bayes factors and the estimated log Bayes factors for different total iteration numbers $N_{iter}$. Figure 6 compares the variance of the log Bayes factor estimates for the three different methods: TI-standard, TI-optimal, and NETI-DIFF. Figure 7 shows ratios of the variances obtained with TI-optimal and NETI-DIFF.

For the Radiata data, NETI-DIFF only achieves a slight reduction in the absolute deviation (Fig. 3) and the variance (Figs. 6, 7) for the lowest number of iterations, $N_{iter}=64k$; otherwise NETI-DIFF and TI-optimal are on a par. Note that the two alternative linear regression models applied to the Radiata data only share the intercept, while their sets of covariables are disjunct. This lack of model overlap presents the least favourable scenario for NETI-DIFF, and our results confirm that there is little room for improvement over standard TI.

For the Pima Indians data, NETI-DIFF achieves a significant reduction in the absolute deviation (Fig. 4) and the variance (Figs. 6, 7) and clearly outperforms both TI methods: TI-standard and TI-optimal. The variance reduction ranges between ratios of 5 and 50. As opposed to the models applied to the Radiata data, the alternative logistic regression models applied to the Pima Indians data are nested, with the parameters of the less complex model forming a subset of those of the more complex one. Our results demonstrate that in this scenario, the new thermodynamic integration path of NETI-DIFF has potential for significant improvement over the established TI methods.

**Fig. 5:**
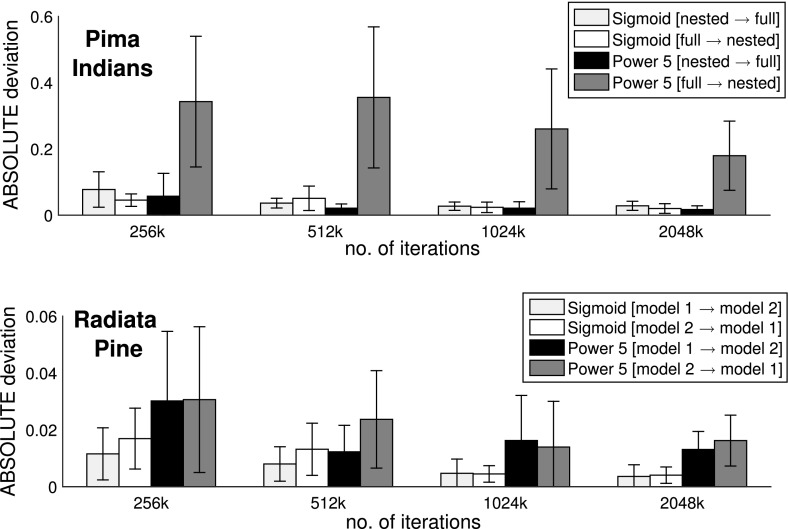
Comparison of two inverse temperature ladders and two NETI-DIFF paths. The *vertical bars* show the average absolute deviations between the estimated and true log Bayes factor, with *error bars* representing standard deviations. The *horizontal axes* give the total number of MCMC iterations $N_{iter}$. The two inverse temperature ladders compared are the power law, Eq. (8), versus the sigmoid function, defined in Sect. 3.6. The alternative NETI-DIFF path swaps the initial model at $\tau =0$ with the final model at $\tau =1$. *Top row:* Radiata pine data. *Bottom row:* Pima Indians data

We also investigated the effect of the inverse temperature ladder (’sigmoid’ vs. ’power 5’) and the starting point (${\mathcal {M}}_1$ vs. ${\mathcal {M}}_2$). To this end, we systematically applied the proposed NETI-DIFF approach with all four combinations (two inverse temperature ladders times two starting points) to the two data sets: Radiata pine and Pima Indians. The results can be found in Fig. 5. First, consider the Pima Indians data, where the two alternative models are nested, and the power inverse temperature scheme of Eq. (8) has been applied. There is a clear advantage of starting the thermodynamic integration at the less complex model over starting at the more complex model: the absolute errors are significantly higher in the latter case. This is not surprising. It is well known from standard TI for computing marginal likelihoods that for the power law of Eq. (8), the optimal transition path is from the prior to the posterior, with the majority of the inverse temperature points at the prior end. Applying this principle to NETI-DIFF, starting the transition path for the differential parameters (i.e. the parameters that are *only* in the more complex model) at the prior, implies that the overall inverse temperature transition path has to lead from the less complex to the more complex model, in confirmation of our findings. Interestingly, for the sigmoid temperature ladder from Sect. 3.6, the difference between the two directions is substantially reduced, which is a natural consequence of the symmetry inherent in this scheme. There is no significant performance difference between the sigmoid and the power law inverse temperature paths when the models are nested (Pima Indians data, top row in Fig. 5). For the Radiata pine data on the other hand (bottom row in Fig. 5), where the alternative models are not nested, the power law of Eq. (8) is intrinsically suboptimal,^10^ and the sigmoidal inverse temperature path of Sect. 3.6 is to be preferred.

### Radiocarbon dating

Next, we consider model selection amongst different polynomial orders for polynomial regression on the Radiocarbon data. Since this is a linear model, the log Bayes factor is known and can be used for evaluating the accuracy of the different thermodynamic integration schemes. Besides comparing the proposed NETI-DIFF scheme with the established TI methods, we investigate the influence of the inverse temperature ladder and the transition path. Due to the comparatively low computational costs, we have increased the number of discretisation points from $K \in \{10,20,50,100\}$ to $K \in \{20,50,100,200\}$.

Figure 8 shows the absolute error (see Eq. 41) for NETI-DIFF and the better of the two established TI methods: TI-optimal. The task is to compute the log Bayes factor for the pairwise comparison of various polynomial orders, as indicated by the horizontal axis of each panel. It turns out that for TI-optimal, the accuracy of the estimate deteriorates with increasing difference of the model orders (black bars in the top panels of Fig. 8), while NETI-DIFF is unaffected by model choice.^11^ In addition, NETI-DIFF considerably outperforms TI-optimal for the lower iteration numbers, as again seen from the top row in Fig. 8.

**Fig. 6:**
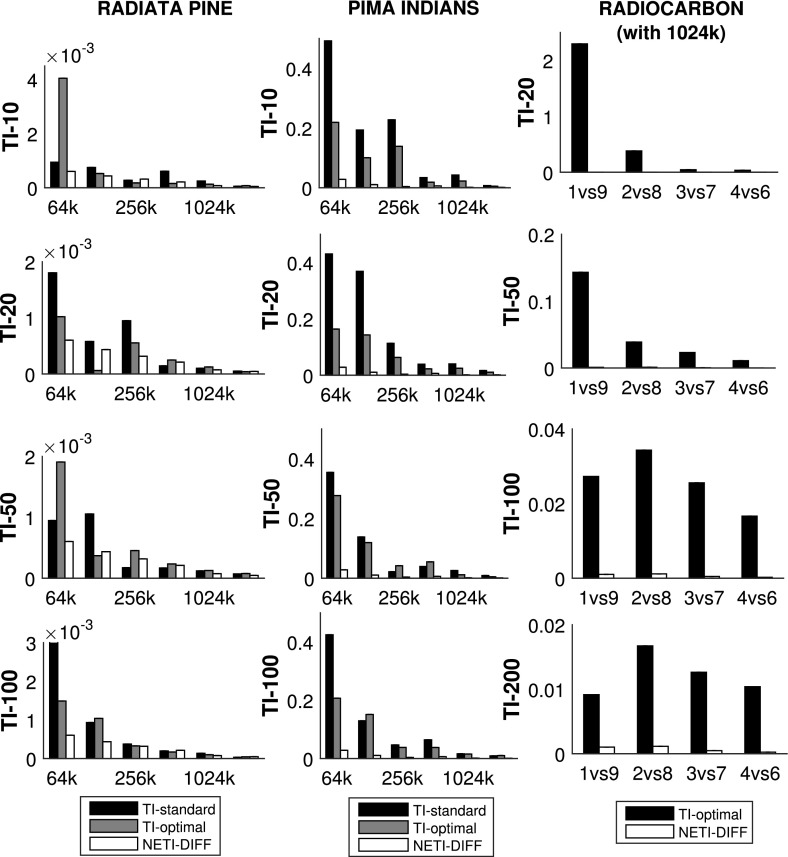
Variance of log Bayes factor estimators. *Left panel:* Radiata pine data, *centre panel:* Pima Indians data, *right panel:* Radiocarbon data. The *vertical bars* show the variance ${\mathbb {V}}$, Eq. (40), for the TI-standard, TI-optimal and NETI-DIFF estimators of the log Bayes factor. For the Radiata pine and the Pima Indians data we varied the number of total MCMC iteration (*horizontal axes*). For the Radiocarbon data we performed $N_{iter}=1024k$ iterations and considered four different pairwise model comparisons (*horizontal axis*). The rows represent different numbers of discretisation points for TI (NETI-DIFF is unaffected). The three columns refer to the four panels in Fig. 3 (*right*), Fig. 4 (*center*) and Fig. 8 (*right*). The corresponding ratios of the variances are shown in Fig. 7

**Fig. 7:**
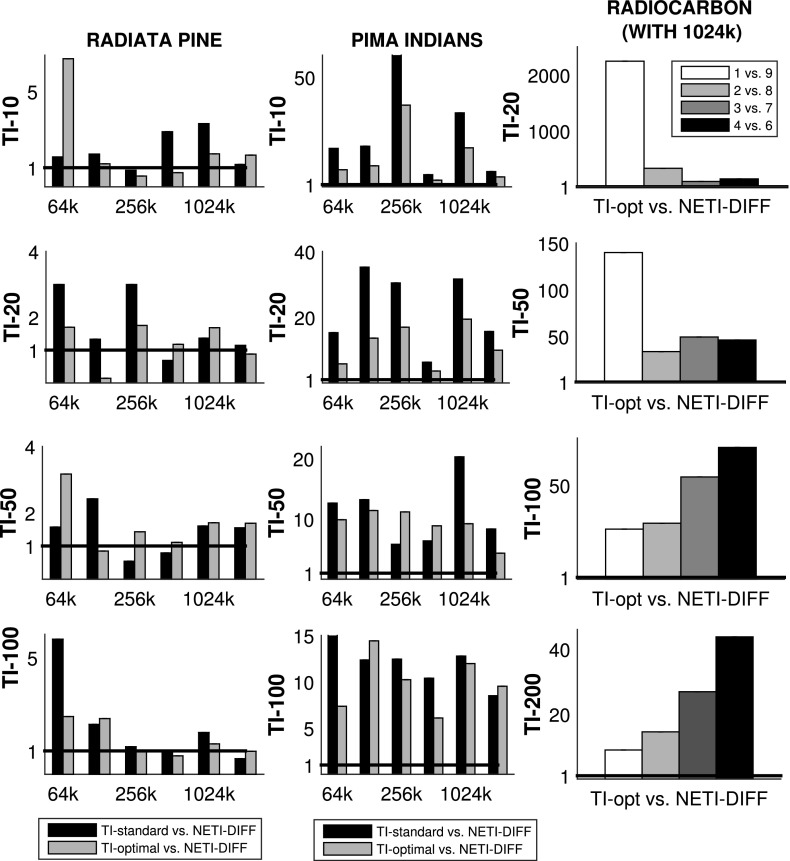
Variance ratios of log Bayes factors estimators. *Left panel:* Radiata pine data, *centre panel:* Pima Indians data, *right panel:* Radiocarbon data. The *vertical bars* show the variance ratios of the log Bayes factor estimators: TI-standard versus NETI-DIFF, and TI-optimal versus NETI-DIFF (obtained from the variances in Fig. 6). The *horizontal reference* line at value 1 indicates equal performance; values above 1 indicate that NETI-DIFF achieves a variance reduction over the established TI schemes. For the Radiata pine and the Pima Indians data we varied the number of total MCMC iterations $N_{iter}$ (*horizontal axes*). For the Radiocarbon data we performed $N_{iter}=1024k$ iterations and considered four different pairwise model comparisons (*horizontal axis*). The rows represent different numbers of discretisation points for TI (NETI-DIFF is unaffected). The three columns refer to the four panels in Fig. 3 (*left*), Fig. 4 (*center*) and Fig. 8 (*right*)

The right column of Fig. 6 compares the variances between NETI-DIFF and TI-optimal, and the right column of Fig. 7 shows the corresponding variance ratios. It is seen that NETI-DIFF consistently outperforms TI-optimal, with the variance ratios ranging between 5 and 2000. It appears that for low iteration numbers $N_{iter}$, the improvement is most pronounced when the alternative models differ substantially (polynomial order 1 vs. 9), while for high iteration numbers $N_{iter}$, the clearest improvement is achieved when the alternative models are more similar (polynomial orders 4 vs. 6).

**Fig. 8:**
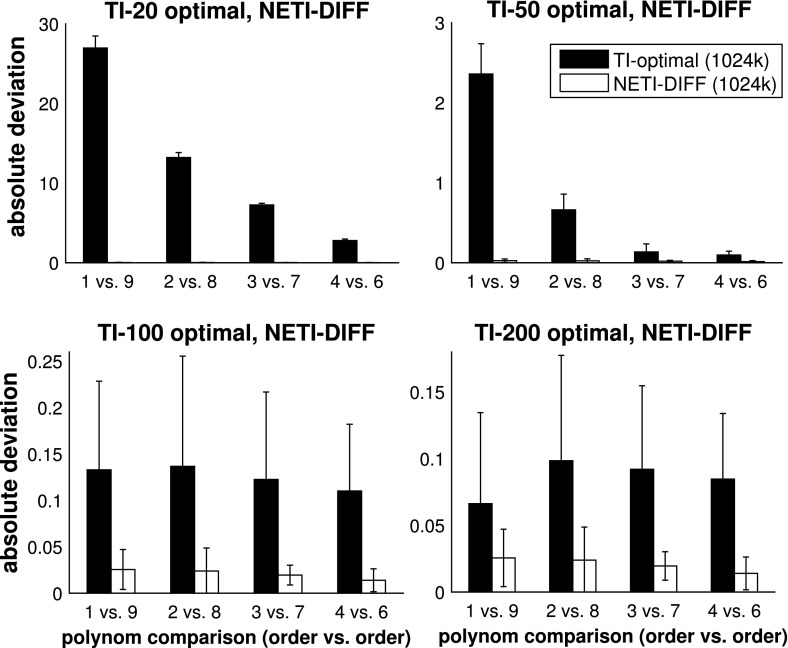
Average absolute error on the Radiocarbon data: NETI versus TI-optimal. The figure shows the average absolute deviation between the estimated and the true log Bayes factor. In each panel the same NETI-DIFF results are shown, while TI-optimal was applied with different numbers of discretisation points (20, 50, 100 and 200). The *bars* represent standard deviations and the *horizontal axes* indicate different model comparisons (polynomials of orders *i* vs. *j*). The total number of (power posterior) MCMC iterations was kept fixed at $N_{iter}=1024k$

**Fig. 9:**
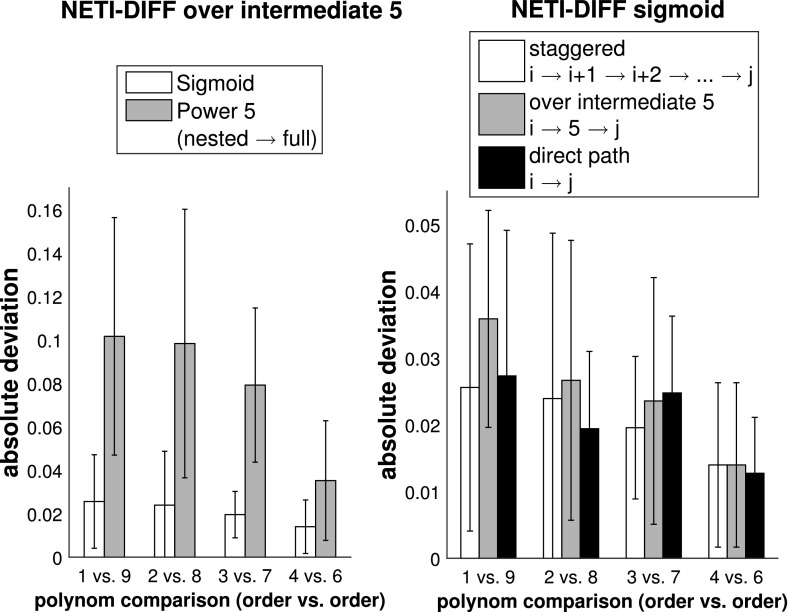
Influence of the inverse temperature ladder and the transition path. The *bars* show the average absolute deviation ${\mathbb {A}}$, Eq. (41), between the estimated and true log Bayes factor, computed with NETI-DIFF for the Radiocarbon data. The *error bars* show standard deviations. The *horizontal axes* indicate different model comparisons (polynomials of orders *i* vs. *j*). The total number of (power posterior) MCMC iterations was kept fixed at $N_{iter}=1024k$. *Left panel:* Comparison of two NETI-DIFF inverse temperature ladders (sigmoid vs. power 5). *Right panel:* Comparison of three NETI-DIFF transition strategies (staggered vs. intermediate vs. direct)

**Fig. 10:**
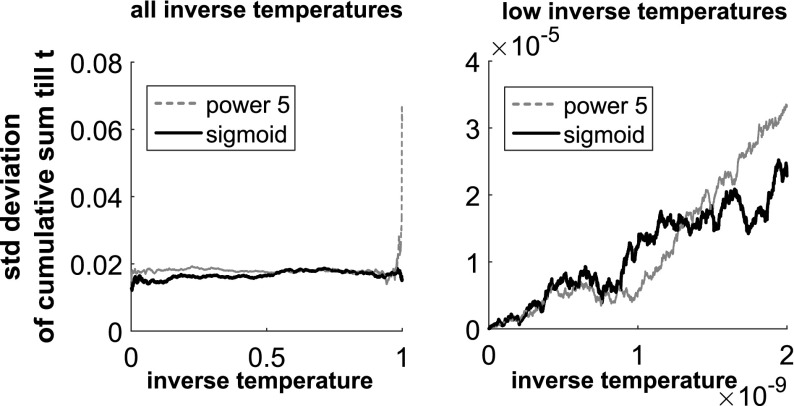
Comparison of the two inverse temperature ladders—Radiocarbon data. The figures show the standard deviation of the partial NETI-DIFF integral, Eq. (18), over the partial inverse temperature range $[0,\tau ]$, obtained from five independent NETI-DIFF simulations. The *right panel* shows a section of the *left panel* at higher resolution. *Dashed line:* power law, Eq. (8). *Solid line:* sigmoid function, defined in Sect. 3.6

The left panel of Fig. 9 compares the two inverse temperature ladders: the power law of Eq. (8) versus the sigmoidal form of Sect. 3.6. Since the models are nested, we would expect the polynomial scheme to perform well, like for the Pima Indians data discussed above. Interestingly, the sigmoidal scheme achieves a better stabilization of the results w.r.t. model order, and a slightly better performance for the largest difference between the polynomial orders of the two alternative models considered. To shed more light on this trend, we have investigated the evolution of the standard deviation of the thermodynamic integral up to a given inverse temperature $\tau$. The results are shown in Fig. 10. While the power law indeed achieves a lower standard deviation than the sigmoidal scheme at the low-inverse-temperature end (near the low-complex model), it contributes a larger proportion to the standard deviation at the high-inverse-temperature end (near the high-complex model). This suggests that the sparsity of inverse temperatures at the high-inverse-temperature end can be counterproductive due to insufficient sample size.

We finally investigated different model transition paths, with a comparison of three alternative schemes: (1) a staggered path from the low-complexity to the high-complexity model via a series of all intermediate models; (2) a transition via one intermediate model of medium complexity; and (3) a direct transition. The results are shown in the right panel of Fig. 9. The differences are small without a clear trend. This suggests that NETI-DIFF is remarkably robust w.r.t. the choice of the model transition path.

### Biopathway

For the biopathway example, we considered two types of data. The first type was obtained from the wild type gene regulatory network shown in Fig. 1a; the second type was obtained from the mutant network shown in Fig. 1b. As we do not have a closed-form expression of the log Bayes factor we chose, as a proxy, the average of the log Bayes factors obtained with the longest TI and NETI-DIFF simulations, which tended to be in reasonably good agreement. Table 1 shows the values of the log Bayes factor thus obtained, which confirms that Bayesian model selection based on the hierarchical model of Fig. 2 consistently identifies the true gene network.

**Table 1:** Log Bayes factor for the biopathway data.

Data instance:	1	2	3	4	5
${{\underline{wildtype}}} \rightarrow$ *PRR7/9*	$-$27.8	$-$29.3	$-$26.1	$-$25.4	$-$28.4
${\underline{PRR7/9}} \rightarrow$ *wildtype*	17.1	9.8	14.4	5.8	4.0

The table shows the log Bayes factor $\log p(D|{\mathcal {M}}_2)/p(D|{\mathcal {M}}_1)$, where ${\mathcal {M}}_1$ is the biopathway from Fig. 1a (wildtype), and ${\mathcal {M}}_2$ is the biopathway from Fig. 1b (PRR7/PRR9 mutant). Top row: data obtained from ${\mathcal {M}}_1$; negative log Bayes factors select the true model. Bottom row: data obtained from ${\mathcal {M}}_2$; positive log Bayes factors select the true model. The five columns show values for different independent data instantiations. The log Bayes factors were obtained by averaging the values obtained with NETI-DIFF and TI for the largest number of iterations $N_{iter}$

**Fig. 11:**
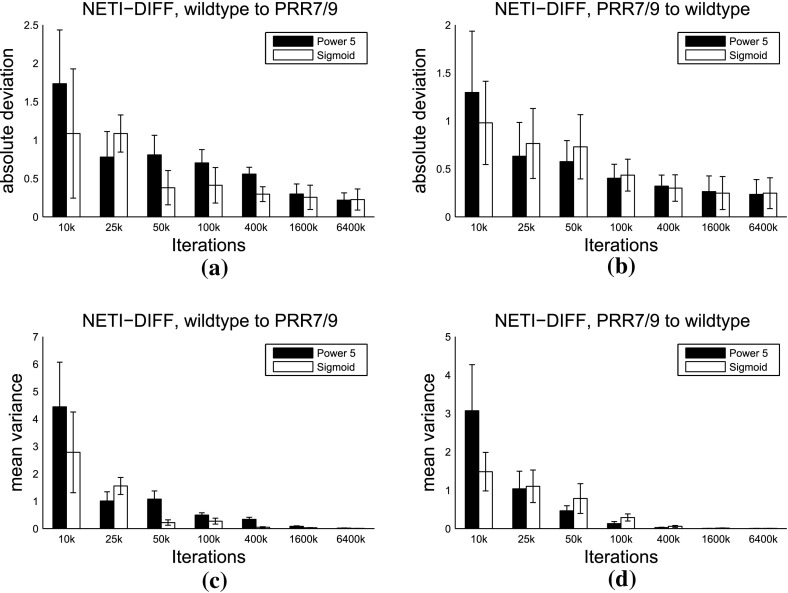
Mean absolute error and mean variance for different inverse temperature ladders and biopathway data. Panels **a**, **b** show a comparison of the mean absolute error ${\mathbb {A}}$ (Eq. 41) and panels **c**, **d** show a comparison of the mean variance ${\mathbb {V}}$ (Eq. 40) between two inverse temperature ladders: the power law from Eq. (8) as *black boxes*, and the sigmoid form from Sect. 3.6 as *white boxes*. Results were obtained from 5 independent data instantiations from the wildtype biopathway of Fig. 1a, and 5 independent data instantiations from the PRR7/PRR9 mutant biopathway of Fig. 1b. Histogram height: average. *Error bars*: standard deviation

In a preliminary study, we compared the two inverse temperature ladders for NETI-DIFF: power law (see Eq. (8)) with power 5, as in Friel et al. (2014), versus the sigmoid transfer function of Sect. 3.6. We repeated the simulations on the 5 data sets of Table 1. From these data sets, we computed the mean of the variance ${\mathbb {V}}$, Eq. (40), and the mean absolute error ${\mathbb {A}}$, Eq. (41). The results are shown in Fig. 11. The trend is not as clear as in Fig. 5. However, the sigmoid inverse temperature ladder achieves more often a performance improvement over the power law (in terms of lower mean absolute error ${\mathbb {A}}$ and average variance ${\mathbb {V}}$) than the other way round, and we therefore adopted it for all subsequent studies.

The main question of interest is to compare TI and NETI-DIFF with respect to accuracy, estimation uncertainty and computational efficiency. To improve the clarity of the presentation, we only show the comparison between NETI-DIFF and TI-optimal, i.e. the TI scheme with the improvements proposed by Friel et al. (2014). In what follows, we refer to “TI-optimal” simply as “TI”. The simulations were repeated for different total iteration lengths, $N_{iter}$, ranging from $N_{iter}=10{,}000$ to $N_{iter}=6{,}400{,}000$ MCMC steps. We repeated TI for different numbers of inverse temperatures, *K*, ranging from $K=10$ to $K=100$ [(the same values as used in Friel et al. (2014)].

**Fig. 12:**
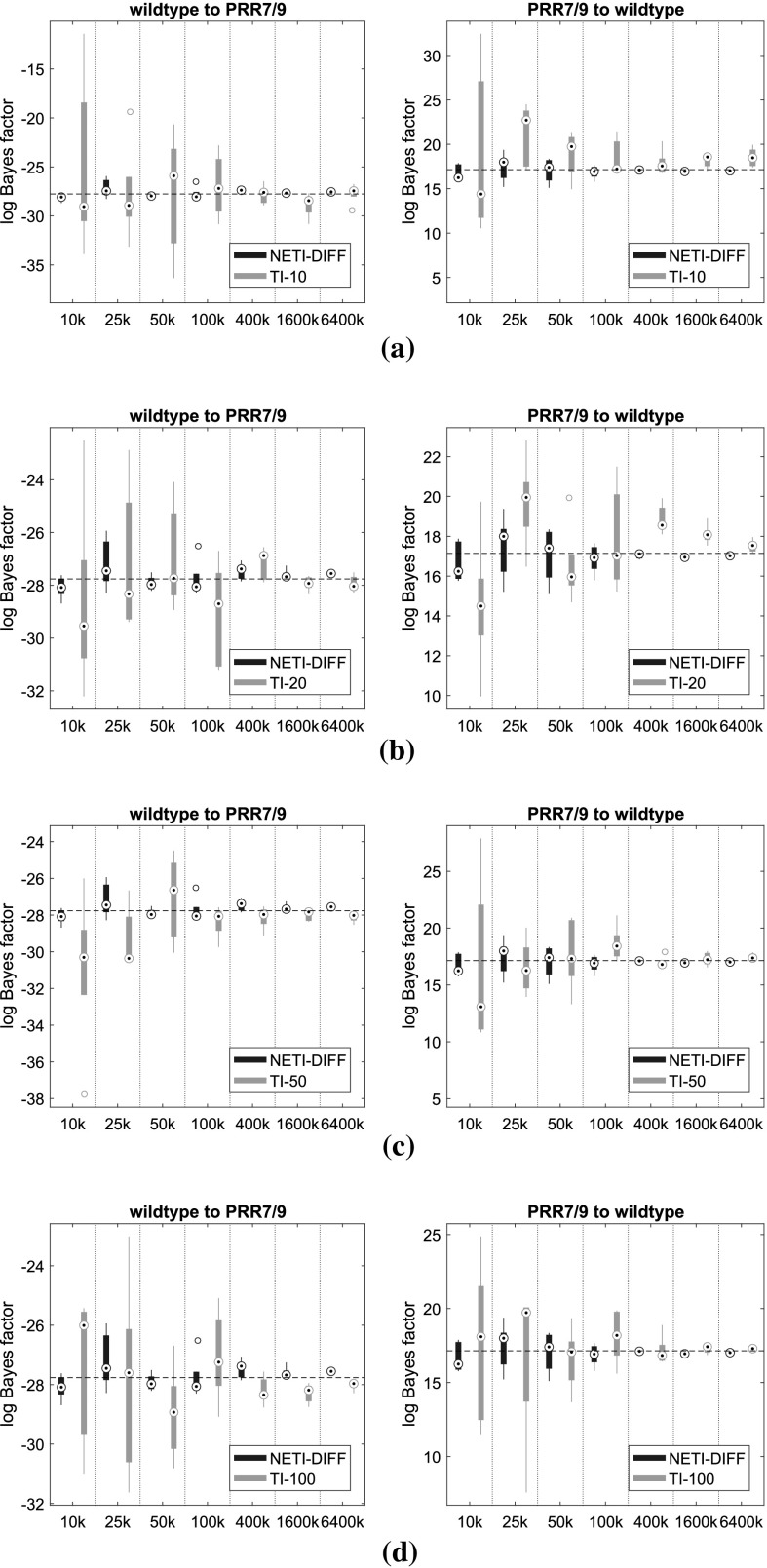
Log Bayes factors for the biopathway data: comparison between NETI-DIFF and TI. The figure shows the distribution of the log Bayes factor $\log p(D|{\mathcal {M}}_2)/p(D|{\mathcal {M}}_1)$, where ${\mathcal {M}}_1$ is the biopathway from Fig. 1a (wildtype), and ${\mathcal {M}}_2$ is the biopathway from Fig. 1b (PRR7/PRR9 mutant). NETI-DIFF is the same for all four rows. Left column: data generated from ${\mathcal {M}}_1$; negative log Bayes factors select the correct model. Right column: data generated from ${\mathcal {M}}_2$; positive log Bayes factors select the correct model. The *horizontal line* shows the ‘true’ value of the log Bayes factor (in the sense defined in the text). The *box plots* show distributions over 5 independent MCMC runs. The *horizontal axis* shows $N_{iter}$, the total number of iterations, ranging from 10 to 6400 k **a** TI with $K=10$ inverse temperatures. **b** TI with $K=20$ inverse temperatures. **c** TI with $K=50$ inverse temperatures. **d** TI with $K=100$ inverse temperatures

**Fig. 13:**
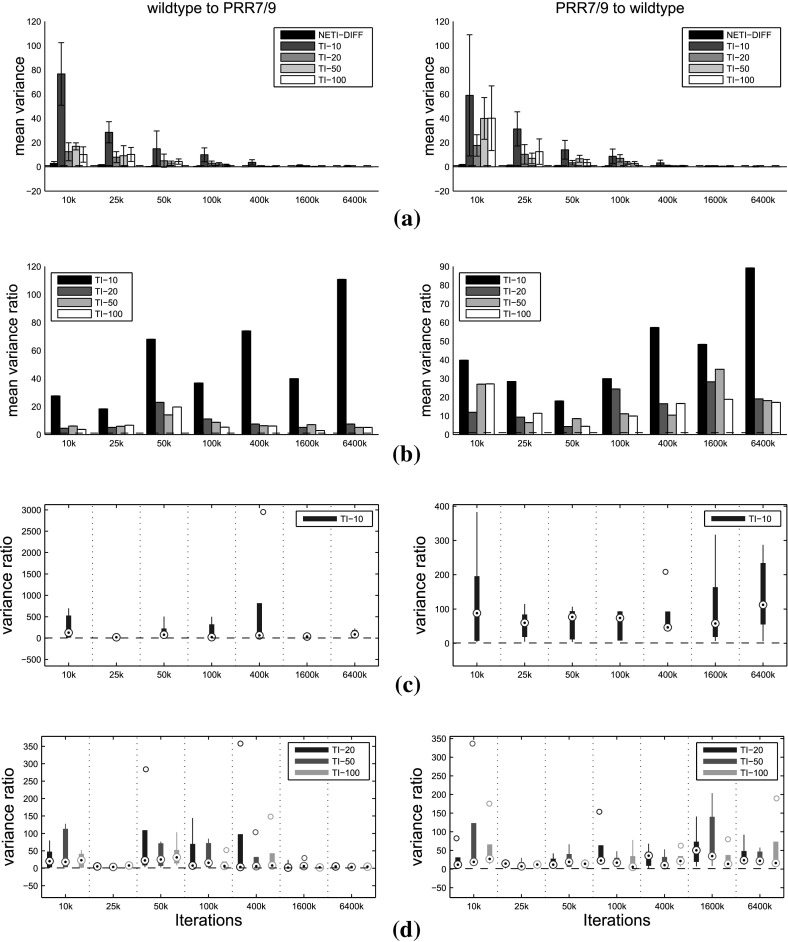
Variance of log Bayes factor estimation: comparison between NET-DIFF and TI on the biopathway data. The variance measures are obtained from five repeated simulations of the same data set. The mean measures correspond to the average from five different data instantiations, obtained from the biopathway of Fig. 1a (wildtype, left column), and from the biopathway of Fig. 1b (PRR7/PRR9 mutant, right column) **a** Mean of variance ${\mathbb {V}}$, defined in Eq. (40) with standard deviations (*error bars*). **b** Ratio of the mean variance obtained with TI, divided by the average variance obtained with NETI-DIFF: ${\overline{{\mathbb {V}}}}(\mathrm {TI})/{\overline{{\mathbb {V}}}}(\mathrm {NETI-DIFF})$. Values above 1 indicate a performance improvement with NETI-DIFF over TI. **c** Distribution of the variance ratios ${\mathbb {V}}(\mathrm {TI})/{\mathbb {V}}(\mathrm {NETI-DIFF})$ for TI with $K=10$ inverse temperatures. Values above 1 indicate a performance improvement with NETI-DIFF over TI. **d** Same as panel c, but for TI with $K=(20,50,100)$ inverse temperatures

**Fig. 14:**
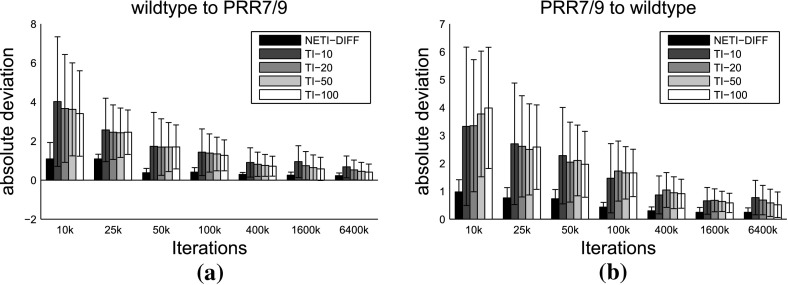
Mean absolute deviation of log Bayes factor estimation: comparison between NETI-DIFF and TI on the biopathway data. Simulations were repeated for 5 independent data instantiations, obtained from the biopathway of Fig. 1a (wild type), and from the biopathway of Fig. 1b (PRR7/PRR9 mutant). Shown is the mean absolute deviation ${\mathbb {A}}$, defined in Eq. (41). *Vertical bar* height: average over the five data instantiations. *Error bars*: standard deviation. The *horizontal axis* shows the total number of iterations $N_{iter}$. For each value of $N_{iter}$, the leftmost bar represents NETI-DIFF. The other *bars* with different *grey shadings* represent TI with different numbers of inverse temperatures, ranging from 10 to 100

Figure 12 shows the distribution of estimated log Bayes factors obtained from $N_{simu}=5$ independent MCMC runs.^12^ The two columns refer to the different data types (from the wild type network, left column, and the mutant network, right column), and the rows (Panels 12a–d) to the number of inverse temperatures used for TI (from $K=10$ to $K=100$; note that NETI-DIFF is unaffected by that choice). The horizontal dashed lines show the ‘true’ value, as described above. As expected, the distribution width tends to decrease with increasing computational costs, $N_{iter}$, and for the highest value, TI and NETI-DIFF tend to be in close agreement, with distributions tightly focused on the ‘true’ values. However, for lower computational costs, $N_{iter} \le 400k$, bias and uncertainty tend to be considerably lower for NETI-DIFF than for TI, irrespective of the number of inverse temperatures used for TI.

For a more systematic investigation, we repeated the MCMC simulations on ten independent data instantiations, for the ten data sets used in Table 1. Five data sets were obtained from the biopathway of Fig. 1a (wildtype), and five data sets were obtained from the biopathway of Fig. 1b (PRR7/PRR9 mutant). For each data set, we computed the mean absolute deviation ${\mathbb {A}}$, defined in Eq. (41), and the variance ${\mathbb {V}}$, as defined in Eq. (40).

The top row in Fig. 13 shows the average variance ${\overline{{\mathbb {V}}}}$, averaged over all data instantiations. The second row shows the ratio of the average variance obtained with TI, divided by the average variance obtained with NETI-DIFF, averaged over all five data instantiations: ${\overline{{\mathbb {V}}}}(\mathrm {TI})/{\overline{{\mathbb {V}}}}(\mathrm {NETI-DIFF})$. The third and fourth rows show the distribution of the variance ratios ${\mathbb {V}}(\mathrm {TI})/{\mathbb {V}}(\mathrm {NETI-DIFF})$ over the five different data instantiations, for different numbers of inverse temperatures (for TI), and different total interation numbers $N_{iter}$. For all ratios, values above 1 indicate a performance improvement with NETI-DIFF over TI. Our results indicate that NETI-DIFF consistently achieves a considerable variance reduction over TI. This reduction is particularly pronounced for small numbers of inverse temperatures, where it reaches up to three orders of magnitude. However, even for the highest number of inverse temperatures the variance reduction NETI-DIFF achieves over TI still varies between one and two orders of magnitude. This clear reduction in estimation uncertainty is matched by a consistent reduction in the estimation error, as quantified in terms of ${\mathbb {A}}$ and shown in Fig. 14. The reduction becomes stronger with decreasing iteration numbers $N_{iter}$ and decreasing numbers of inverse temperatures, which indicates that the performance improvement of NETI-DIFF over TI is particularly relevant in the regime of limited computational resources.

## Discussion

The objective of our work has been the direct targeting of the log Bayes factor via a modified thermodynamic integration path. This has been motivated by statistical physics, where the computation of a reaction free energy (mathematically equivalent to the log Bayes factor) is computationally more efficient than the computation of the difference of standard free energies (equivalent to the difference of log marginal likelihoods). The modified transition path directly connects the posterior distributions of the two models involved. In this way, the high variance prior regime is avoided. We have carried out a comparative evaluation with the state-of-the-art TI method of Friel et al. (2014). Our study confirms that a substantial variance reduction can be achieved when the models to be compared are nested. There is little room for improvement when comparing non-nested models with non-overlapping parameter sets. However, even in this least favourable case, the performance achieved with the proposed method, referred to as NET-DIFF in the present manuscript, is still on a par with established TI methods. For inference in a complex systems described by coupled nonlinear differential equations (biopathway), we found that NETI-DIFF reduces the variance by up to two orders of magnitude over state-of-the-art TI methods. Our work has also revealed that NETI-DIFF achieves a considerable performance stabilisation with respect to a variation of the parameter prior.

When the task is model selection out of a set of cardinality *m*, carrying out direct pairwise comparisons is of computational complexity $m^2$ and may not be viable in practice. However, rather than reverting to the standard TI scheme and computing the marginal likelihoods

42 $$\begin{aligned} p(D|{\mathcal {M}}_1),\ldots ,p(D|{\mathcal {M}}_m) \end{aligned}$$

it appears more sensible to compute the Bayes factors

43 $$\begin{aligned} \frac{p(D|{\mathcal {M}}_1)}{p(D|{\mathcal {M}}_0)},\ldots ,\frac{p(D|{\mathcal {M}}_m)}{p(D|{\mathcal {M}}_0)} \end{aligned}$$

where ${\mathcal {M}}_0$ is a typical or representative model chosen from the set of models compared. The results for the Radiocarbon data, reported in Sect. 5.2, have demonstrated a remarkable robustness of the proposed method w.r.t. a variation of the model transition path, meaning that there is no significant difference in efficiency and accuracy between the direct computation of $\log \frac{p(D|{\mathcal {M}}_1)}{p(D|{\mathcal {M}}_2)}$, and the indirect computation via $\log \frac{p(D|{\mathcal {M}}_1)}{p(D|{\mathcal {M}}_0)}$ and $\log \frac{p(D|{\mathcal {M}}_2)}{p(D|{\mathcal {M}}_0)}$. This suggests that 1-out-of-*m* model selection can also be improved with the method we have proposed. It is beyond the scope of this article to investigate this conjecture at greater depth, but it appears plausible that targeting Bayes factors along an annealing path starting from a reference posterior distribution associated with a reference model should give smaller posterior variance than conventionally targeting marginal likelihoods along an annealing path starting from the prior distribution.

If there are only those *m* models, ${\mathcal {M}}_1,\ldots ,{\mathcal {M}}_m$, then the *m* Bayes factors in Eq. (43) together with the (pre-defined) model prior probabilities $p({\mathcal {M}}_i)$ ($i=1,\ldots ,m$) and the normalisation condition fully specify the model posterior probabilities $p({\mathcal {M}}_i|D)$. With the definition:

$$\begin{aligned} b_{i,j} := \frac{p(D|{\mathcal {M}}_i)}{p(D|{\mathcal {M}}_j)} \cdot \frac{p({\mathcal {M}}_i)}{p({\mathcal {M}}_j)} = \frac{p(D|{\mathcal {M}}_i)}{p(D|{\mathcal {M}}_0)} \cdot \left( \frac{p(D|{\mathcal {M}}_j)}{p(D|{\mathcal {M}}_0)}\right) ^{-1} \cdot \frac{p({\mathcal {M}}_i)}{p({\mathcal {M}}_j)} \;\;\;\;\;\; \left( i,j\in \{1,\ldots ,m\}\right) \end{aligned}$$

where the two Bayes factors on the right are known from Eq. (43), we get:

44 $$\begin{aligned} p({\mathcal {M}}_i|D) = \frac{p(D|{\mathcal {M}}_i)\cdot p({\mathcal {M}}_i)}{\sum _{j=1}^{m} p(D|{\mathcal {M}}_j)\cdot p({\mathcal {M}}_j) } = \frac{p(D|{\mathcal {M}}_i)\cdot p({\mathcal {M}}_i)}{\sum _{j=1}^{m} \frac{p(D|{\mathcal {M}}_i)\cdot p({\mathcal {M}}_i)}{b_{i,j}} } = \left( \sum _{j=1}^m b_{i,j}^{-1} \right) ^{-1} \end{aligned}$$

Equation (44) is formally equivalent to Eq. (4) in Berger and Delampady (1987). We have *m* models with discrete prior probabilities $\pi _i = P({\mathcal {M}}_i)>0$ and $\sum _{i=1}^m \pi _i=1$. We get, e.g., for model ${\mathcal {M}}_1$:

$$\begin{aligned} p({\mathcal {M}}_1|D)= & {} \left( 1 + \sum _{j=2}^m b_{1,j}^{-1} \right) ^{-1} = \left( 1 + \sum _{j=2}^m \frac{\pi _j}{\pi _1} \cdot \frac{p(D|{\mathcal {M}}_j)}{p(D|{\mathcal {M}}_1)} \right) ^{-1} \\= & {} \left( 1 + \frac{1}{\pi _1 } \sum _{j=2}^m \pi _j \cdot \frac{p(D|{\mathcal {M}}_j)}{p(D|{\mathcal {M}}_1)} \right) ^{-1} = \left( 1 + \frac{1-\pi _1}{\pi _1} \sum _{j=2}^m \frac{\pi _j}{1-\pi _1} \cdot \frac{p(D|{\mathcal {M}}_j)}{p(D|{\mathcal {M}}_1)} \right) ^{-1}\\= & {} \left( 1 + \frac{1-\pi _1}{\pi _1} \cdot \frac{\sum _{j=2}^m p(D|{\mathcal {M}}_j) \cdot \frac{\pi _j}{1-\pi _1}}{p(D|{\mathcal {M}}_1)} \right) ^{-1} = \left( 1 + \frac{1-\pi _1}{\pi _1} \cdot \frac{1}{B} \right) ^{-1} \end{aligned}$$

where *B* is the Bayes factor:

45 $$\begin{aligned} B:=\frac{p(D|{\mathcal {M}}_1)}{\sum _{j=2}^m p(D|{\mathcal {M}}_j)\cdot g({\mathcal {M}}_j)} = \frac{p(D|{\mathcal {H}}_0)}{p(D|{\mathcal {H}}_1)} \;\; with \;\; g({\mathcal {M}}_j):=\frac{\pi _j}{1-\pi _1} = \frac{\pi _j}{\sum _{j=2}^m \pi _j} \end{aligned}$$

and the hypotheses stand for: ${\mathcal {H}}_0{:}{\mathcal {M}}={\mathcal {M}}_1$ and ${\mathcal {H}}_1{:}{\mathcal {M}}\in \{{\mathcal {M}}_2,\ldots ,{\mathcal {M}}_m\}$ which are assumed to be true with the prior probabilities $\pi _1$ and $1-\pi _1$, respectively.  Equation (45) corresponds to Eq. (2) in Berger and Delampady (1987).^13^

One of the referees raised the interesting question of how the proposed method is applied to graphical Gaussian models and mixture models.

We have included an additional section in the Appendix 7.4 where we discuss in detail how the proposed method can be applied to Graphical Gaussian models. We have also carried out an additional simulation study to illustrate the application of our method to Graphical Gaussian models. The key idea is to not apply the method to the configuration space of precision matrices directly, which would be cumbersome due to the constrained topology of this space (restriction to positive definite matrices). Instead, we make use of the theorem that every multivariate normal density can be represented by a Gaussian belief network, and vice versa; see Geiger and Heckerman (1994). This effectively defines an isomorphism between the space of Gaussian graphical models and the space of Gaussian belief networks. We exploit this isomorphism by defining the proposed NETI scheme in the space of Gaussian belief networks, as discussed in detail in Appendix 7.4.

For mixture models, the proposed NETI method will not achieve any improvement over the standard thermodynamic integration scheme. The reason is that according to Eq. (18), the modified thermodynamic integration path that we have proposed has the potential for a variance reduction if the two model likelihoods in the numerator and denominator share a substantial number of parameters. For mixture models, this is not the case, due to the intrinsic identifiability problem. In Appendix 7.5, we demonstrate on an empirical simulation study that for a mixture model, the proposed new method and the established thermodynamic integration scheme are on a par.

The focus of our study has been a comparison with the improved TI method proposed in Friel et al. (2014). Recently, a powerful new method for variance reduction in thermodynamic integration based on control variates, termed CTI (controlled thermodynamic integral), has been proposed (Oates et al. 2016). The idea is to add a zero-mean function from a given function family (e.g. a polynomial) to the integrand and then apply variational calculus to minimise the variance of the estimator. The resulting optimality equations depend on expectation values w.r.t. the unknown posterior distribution, which the authors approximate with samples from initial MCMC simulations.

On the Radiata data, CTI outperforms NETI-DIFF, due to the fact that NETI-DIFF offers little room for improvement on non-nested models with disjunct parameter sets, as discussed above. On the Pima Indians data, both NETI-DIFF and CTI achieve a significant variance reduction over the state-of-the-art TI method of Friel et al. (2014). Oates et al. (2016) applied their method with the standard trapezoid sum of Eq. (7), CTI-1, and with the improved trapezoid sum of Eq. (10), CTI-2. A comparison between Fig. 7 in the present paper and Fig. 3 in Oates et al. (2016) shows that the performance of NETI-DIFF, which reduces the variance over state-of-the-art TI by a whole order of magnitude, lies between CTI-1 and CTI-2. Oates et al. (2016) argue that the linear curvature sum of Eq. (7) is known to be biased, and the quadratic curvature rule of Eq. (10) should be used. However, in Aderhold et al. (2017) it was demonstrated that quadratic curvature can lead to an increase in the estimation error when vague prior distributions are used, and it is therefore not always the automatic method of choice.

Current work in statistics is increasingly aiming to tackle more complex models, e.g. based on coupled nonlinear differential equations, like the biopathway model discussed in Sect. 4.4. For data generated from an ordinary differential equation model of circadian regulation (Goodwin oscillator), Oates et al. (2016) found that CTI achieved little improvement over state-of-the-art TI. The authors discuss that a potential problem CTI faces for complex models is multimodality of the posterior distributions, rendering the approximation of the posterior expectation values, which enter the optimality equations from variational calculus, less reliable. NETI-DIFF, on the other hand, does not rely on such estimates. In fact, our results, presented in Fig. 13, suggest that NETI-DIFF achieves the most substantial variance reduction over state-of-the-art TI for the most complex, nonlinear biopathway model, reaching up to and exceeding two orders of magnitude.

We conclude that CTI and NETI-DIFF are *not* competing methods, but rather conceptionally different approaches with the potential to complement each other. CTI aims to achieve variance reduction by adding control variates to the integrand; it requires a reliable estimation of posterior averages of quantities related to these control variates from initial MCMC runs. NETI-DIFF aims to achieve variance reduction by modifying the thermodynamic integration path; it works best for models with substantial parameter overlap. Both approaches can be combined, that is, the natural next step is to add control variates *and* change the integration path, i.e. to target the log Bayes factor with the principles of CTI. This combination of NETI-DIFF and CTI has the potential to further extend the feasibility of Bayesian model selection to ever more complex models, and a closer investigation of such a hybrid approach poses a promising avenue for future research.

## Figures and Tables

**Fig. 15:**
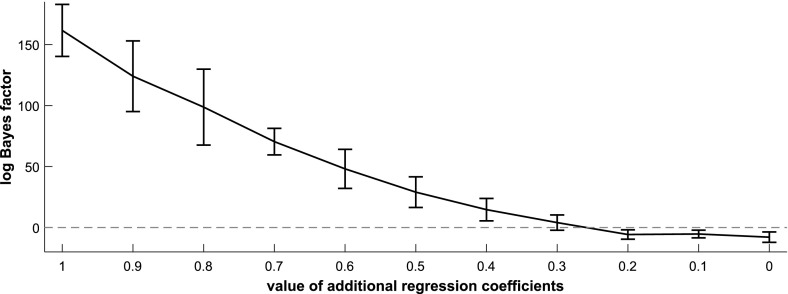
Average log Bayes factor for GGMs: wildtype (${\mathcal {M}}_1$) versus mutant (${\mathcal {M}}_2$) network. The figure shows the average log Bayes factor obtained for 10 independent wildtype data instantiations with $n=100$ observations each. Bayes factors above the dotted reference line are in favour of the wildtype network (${\mathcal {M}}_1$), which is the true network if the additional regression coefficients are greater than 0. The *error bars* refer to standard deviations

**Fig. 16:**
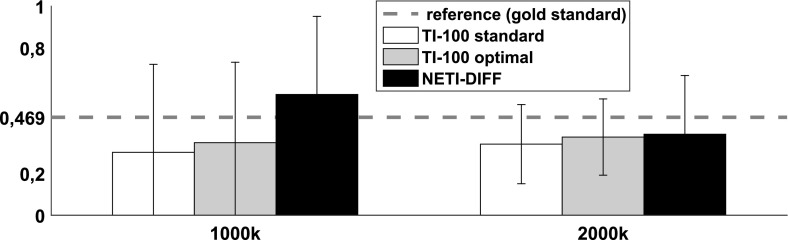
Bayes factor estimates for the Galaxy data. *Error bar* plot of the average log Bayes factor between the mixture models ${\mathcal {M}}_4$ with $K=4$ components and ${\mathcal {M}}_3$ with $K=3$ components. For each method the averages have been computed from 10 independent simulations. The *dotted reference line* indicates the (true) gold-standard Bayes factor of size 0.4685, reported in Friel et al. (2014). The *error bars* correspond to standard deviations, and the *horizontal axis* gives the total number of iterations ($N_{iter}=1000k$ and $N_{iter}=2000k$)

**Fig. 17:**
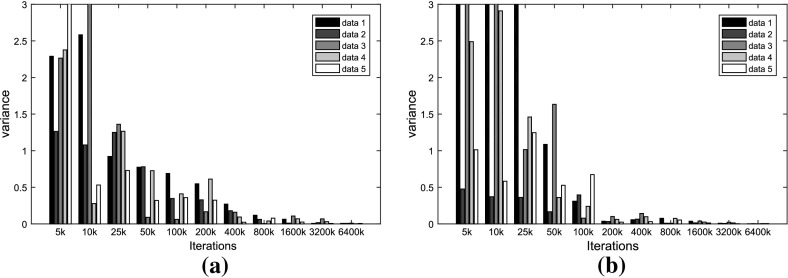
Convergence of NETI-DIFF for the Biopepa data. This figure gives the variance of marginal log likelihood estimators for NETI-DIFF and the two ladder types (panel a and b). The variance is calculated from five repetitions and for five different data instances shown in the *legend*. This figure complements Fig. 11, where the average variances, averaged over all five data instantiations, are shown. **a** NETI-DIFF with power law ladder. **b** NETI-DIFF with sigmoidal ladder

## Appendix

### Proof of Jarzynski’s theorem

Using the definitions from Sect. 2, we get:

$$\begin{aligned} p(D|{\mathcal {M}}_i)= & {} \int p(D|{\varvec{\theta }},{\mathcal {M}}_i)p({\varvec{\theta }}) d{\varvec{\theta }} = \frac{ \int \exp (-E_i[{\varvec{\theta }}]) p({\varvec{\theta }}) d{\varvec{\theta }} }{ \int p({\varvec{\theta }}) d{\varvec{\theta }} } \\= & {} \frac{\int \exp (-E_i[{\varvec{\theta }}]) p({\varvec{\theta }}) d{\varvec{\theta }}}{ \int \exp (E_i[{\varvec{\theta }}]) \exp (-E_i[{\varvec{\theta }}]) p({\varvec{\theta }}) d{\varvec{\theta }}} \\= & {} \left( \int \exp (E_i[{\varvec{\theta }}]) \frac{\exp (-E_i[{\varvec{\theta }}]) p({\varvec{\theta }}) d{\varvec{\theta }}}{\int \exp (-E_i[{\varvec{\theta }}]) p({\varvec{\theta }}) d{\varvec{\theta }}} \right) ^{-1}\\= & {} \left( \int \exp (E_i[{\varvec{\theta }}]) p({\varvec{\theta }}|D,{\mathcal {M}}_i) \right) ^{-1} \\= & {} \Big \langle \exp (E_i[{\varvec{\theta }}]) \Big \rangle _i^{-1} \\ p(D|{\mathcal {M}}_2)= & {} \int \exp (-E_2[{\varvec{\theta }}]) p({\varvec{\theta }}|{\mathcal {M}}_2) d{\varvec{\theta }} \\= & {} \int \exp (-\{E_2[{\varvec{\theta }}]-E_1[{\varvec{\theta }}]\}) \exp (-\{E_1[{\varvec{\theta }}]\}) p({\varvec{\theta }}|{\mathcal {M}}_2) d{\varvec{\theta }} \\= & {} \int \exp (-\varDelta E[{\varvec{\theta }}]) \exp (-\{E_1[{\varvec{\theta }}]\}) p({\varvec{\theta }}|{\mathcal {M}}_2) d{\varvec{\theta }} \\= & {} \int \exp (-\varDelta E[{\varvec{\theta }}]) \exp (-\{E_1[{\varvec{\theta }}]\}) \frac{p({\varvec{\theta }}|{\mathcal {M}}_2)}{p({\varvec{\theta }}|{\mathcal {M}}_1)} p({\varvec{\theta }}|{\mathcal {M}}_1)d{\varvec{\theta }} \\= & {} \int \exp (-\varDelta {\tilde{E}}[{\varvec{\theta }}]) \exp (-\{E_1[{\varvec{\theta }}]\}) p({\varvec{\theta }}|{\mathcal {M}}_1)d{\varvec{\theta }} \\ \frac{p(D|{\mathcal {M}}_2)}{p(D|{\mathcal {M}}_1)}= & {} \int \exp (-\varDelta {\tilde{E}}[{\varvec{\theta }}]) \frac{\exp (-\{E_1[{\varvec{\theta }}]\}) p({\varvec{\theta }}|{\mathcal {M}}_1)}{p(D|{\mathcal {M}}_1)} d{\varvec{\theta }} \\= & {} \int \exp (-\varDelta {\tilde{E}}[{\varvec{\theta }}]) p({\varvec{\theta }}|D,{\mathcal {M}}_1) d{\varvec{\theta }} \\= & {} \Big \langle \exp (-\varDelta {\tilde{E}}[{\varvec{\theta }}]) \Big \rangle _1 \end{aligned}$$

### Uncertainty quantification

From Eq. (16) we have:

46 $$\begin{aligned}&\frac{d}{d \tau }{\mathbb {E}}_{\tau }\left[ \log \left( \frac{p(D|{\varvec{\theta }},{\mathcal {M}}_2)}{p(D|{\varvec{\theta }},{\mathcal {M}}_1)}\right) \right] \nonumber \\&\quad = \frac{d}{d \tau } \left( \frac{1}{Z(D|\tau ,{\mathcal {M}}_1,{\mathcal {M}}_2)} \frac{d}{d\tau } Z(D|\tau ,{\mathcal {M}}_1,{\mathcal {M}}_2) \right) \nonumber \\&\quad = \frac{1}{Z(D|\tau ,{\mathcal {M}}_1,{\mathcal {M}}_2)} \frac{d^2}{d\tau ^2} Z(D|\tau ,{\mathcal {M}}_1,{\mathcal {M}}_2) \nonumber \\&\qquad - \left( \frac{1}{Z(D|\tau ,{\mathcal {M}}_1,{\mathcal {M}}_2)} \frac{d}{d\tau } Z(D|\tau ,{\mathcal {M}}_1,{\mathcal {M}}_2) \right) ^2 \nonumber \\&\quad = \frac{1}{Z(D|\tau ,{\mathcal {M}}_1,{\mathcal {M}}_2)} \frac{d^2}{d\tau ^2} Z(D|\tau ,{\mathcal {M}}_1,{\mathcal {M}}_2) - \left\{ {\mathbb {E}}_{\tau }\left[ \log \left( \frac{p(D|{\varvec{\theta }},{\mathcal {M}}_2)}{p(D|{\varvec{\theta }},{\mathcal {M}}_1)}\right) \right] \right\} ^2\nonumber \\ \end{aligned}$$

For the first term we get:

47 $$\begin{aligned}&\frac{1}{Z(D|\tau ,{\mathcal {M}}_1,{\mathcal {M}}_2)} \frac{d^2}{d\tau ^2} Z(D|\tau ,{\mathcal {M}}_1,{\mathcal {M}}_2) \nonumber \\&\quad = \frac{1}{Z(D|\tau ,{\mathcal {M}}_1,{\mathcal {M}}_2)} \int \frac{d^2}{d\tau ^2} \left( \frac{p(D|{\varvec{\theta }},{\mathcal {M}}_2)}{p(D|{\varvec{\theta }},{\mathcal {M}}_1)} \right) ^{\tau } p(D|{\varvec{\theta }},{\mathcal {M}}_1) p({\varvec{\theta }}|{\mathcal {M}}_1,{\mathcal {M}}_2) d{\varvec{\theta }} \nonumber \\&\quad = \int \left\{ \log \left( \frac{p(D|{\varvec{\theta }},{\mathcal {M}}_2)}{p(D|{\varvec{\theta }},{\mathcal {M}}_1)} \right) \right\} ^2 \frac{p(D|{\varvec{\theta }},{\mathcal {M}}_2)^{\tau } p(D|{\varvec{\theta }},{\mathcal {M}}_1)^{1-\tau } p({\varvec{\theta }}|{\mathcal {M}}_1,{\mathcal {M}}_2)}{Z(D|\tau ,{\mathcal {M}}_1,{\mathcal {M}}_2)} d{\varvec{\theta }} \nonumber \\&\quad = \int p_{\tau }({\varvec{\theta }}|D,{\mathcal {M}}_1,{\mathcal {M}}_2) \left\{ \log \left( \frac{p(D|{\varvec{\theta }},{\mathcal {M}}_2)}{p(D|{\varvec{\theta }},{\mathcal {M}}_1)} \right) \right\} ^2 d{\varvec{\theta }} \nonumber \\&\quad = {\mathbb {E}}_{\tau }\left[ \left\{ \log \left( \frac{p(D|{\varvec{\theta }},{\mathcal {M}}_2)}{p(D|{\varvec{\theta }},{\mathcal {M}}_1)}\right) \right\} ^2 \right] \end{aligned}$$

Combining Eqs. (46) and (47), we get:

$$\begin{aligned} \frac{d}{d \tau }{\mathbb {E}}_{\tau }\left[ \log \left( \frac{p(D|{\varvec{\theta }},{\mathcal {M}}_2)}{p(D|{\varvec{\theta }},{\mathcal {M}}_1)}\right) \right] = {\mathbb {V}}_{\tau } \left[ \log \left( \frac{p(D|{\varvec{\theta }},{\mathcal {M}}_2)}{p(D|{\varvec{\theta }},{\mathcal {M}}_1)}\right) \right] \end{aligned}$$

Define the following shorthand notation:

48 $$\begin{aligned} \varPhi (\tau ) = \left\{ \log \left( \frac{p(D|{\varvec{\theta }},{\mathcal {M}}_2)}{p(D|{\varvec{\theta }},{\mathcal {M}}_1)}\right) \right\} _{\tau } \end{aligned}$$

which is an estimator of ${\mathbb {E}}_{\tau }\left[ \log \left( \frac{p(D|{\varvec{\theta }},{\mathcal {M}}_2)}{p(D|{\varvec{\theta }},{\mathcal {M}}_1)}\right) \right]$ with sample size 1. We can rewrite Eq. (18) as:

49 $$\begin{aligned} \log \left( \frac{p(D|{\mathcal {M}}_2)}{p(D|{\mathcal {M}}_1)}\right)\approx & {} \int _0^1 \varPhi (\tau ) d \tau \; \approx \; \sum _n \varPhi (\tau _n) \varDelta \tau _n \end{aligned}$$

For the variance we get:

50 $$\begin{aligned} {\mathbb {V}}\left\{ \log \left( \frac{p(D|{\mathcal {M}}_2)}{p(D|{\mathcal {M}}_1)}\right) \right\}\approx & {} \sum _n {\mathbb {V}}\Big [\varPhi (\tau _n)\Big ] [\varDelta \tau _n]^2 \;\approx \; \sum _n \left( \frac{\partial \varPhi }{\partial \tau } \right) _{\tau _n}\, [\varDelta \tau _n]^2\nonumber \\\approx & {} \sum _n \frac{\varDelta \varPhi (\tau _n)}{\varDelta \tau _n} [\varDelta \tau _n]^2 \end{aligned}$$

51 $$\begin{aligned}\approx & {} \sum _n \varDelta \varPhi (\tau _n) \varDelta \tau _n \end{aligned}$$

### Pseudocode

Table 2 shows the NETI-DIFF pseudocode for the Bayesian hierarchical model of Fig. 2. Pseudocode for standard MCMC, following a Metropolis-Hastings within Gibbs scheme, was provided in Table 1 of Aderhold et al. (2017). Table 2 shows the modification required to sample with the NETI-DIFF scheme from the tempered posterior distribution in Eq. (13).

**Table 2:** Pseudo code for a NETI-DIFF simulation at inverse temperature $\tau$ for the biopathway model from Sect. 4.4

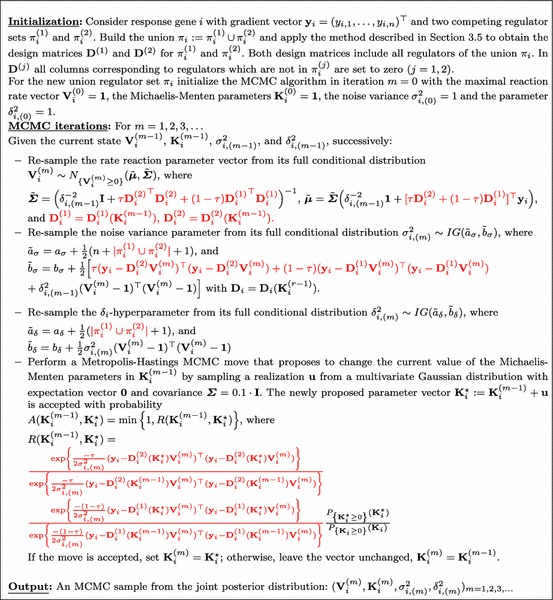

The differences to the standard MCMC scheme from Table 1 in Aderhold et al. (2017) are marked in *red fonts*

### Application to Gaussian Graphical Models

In this Appendix we show how the new method (NETI-DIFF) can be used to infer the Bayes factor between Gaussian graphical models (GGMs). We propose an indirect procedure which exploits that multivariate Gaussians can be represented as ’Gaussian belief networks’ (Geiger and Heckerman 1994). A Gaussian graphical model corresponds to an $M$-dimensional multivariate Gaussian distribution with mean vector ${\mathbf{m}}$ and covariance matrix ${{\varvec{\varSigma }}}$ so that the density (PDF) is given by

52 $$\begin{aligned} p({\mathbf{x}}|{\mathbf{m}},{\mathbf{W}}) = \left( 2\pi \right) ^{-M/2} \cdot \det ({\mathbf{W}}) \cdot \exp \{- \frac{1}{2} ({\mathbf{x}}-{\mathbf{m}})^{{}^{\mathrm{T}}} {\mathbf{W}} ({\mathbf{x}}-{\mathbf{m}}) \} \end{aligned}$$

where ${\mathbf{x}}=(x_1,\ldots ,x_M)^{{}^{\mathrm{T}}}$ and ${\mathbf{W}}={{\varvec{\varSigma }}}^{-1}$ is called the precision matrix. Each 0 element of ${\mathbf{W}}$ indicates that the partial correlation between the corresponding variables is zero, e.g. ${\mathbf{W}}_{i,j}=0$ if the partial correlation between $x_i$ and $x_j$ is zero. We follow Geiger and Heckerman (1994) and identify this Gaussian distribution with a ’Gaussian belief network’, i.e. we factorise the density in Eq. (52) with the chain rule:

53 $$\begin{aligned} p({\mathbf{x}}|{\mathbf{m}},{\mathbf{W}}) = p(x_1) \cdot \prod _{i=1}^{M} p(x_i|x_1,\ldots ,x_{i-1}) \end{aligned}$$

where the conditional distributions are univariate Gaussians

54 $$\begin{aligned} x_i|x_1,\ldots ,x_{i-1} \sim {\mathcal {N}}(m_i+\sum _{j=1}^{i-1} \beta _{j,i} (x_j-m_j),\sigma ^2_i) \end{aligned}$$

${\mathbf{W}}_{j,i}=0$ implies that the ’regression coefficient’ $\beta _{j,i}$ of the Gaussian belief network representation is zero, and vice-versa. Moreover, we have ${\mathbf{m}}=(m_1,\ldots ,m_{M})^{{}^{\mathrm{T}}}$, and $\sigma _i^2$ is the conditional variance of $x_i$ given $x_1,\ldots ,x_{i-1}$. From the parameters in Eqs. (53, 54) the precision matrix ${\mathbf{W}}={\mathbf{W}}(M)$ of the multivariate Gaussian distribution can be (re-)computed with the recursion:

55 $$\begin{aligned} {\mathbf{W}}(i+1) = \left( \begin{matrix} {\mathbf{W}}(i) + \frac{{{\varvec{\beta }}}_i {{\varvec{\beta }}}_i^{{}^{\mathrm{T}}}}{\sigma _{i+1}^2} &{}\quad - \frac{{{\varvec{\beta }}}_i}{\sigma _{i+1}^2} \\ - \frac{{{\varvec{\beta }}}_i^{{}^{\mathrm{T}}}}{\sigma _{i+1}^2} &{}\quad \frac{1}{\sigma _{i+1}^2} \end{matrix} \right) \end{aligned}$$

where $${\mathbf{W}}(1)=\frac{1}{\sigma _1^2}$$ and $${{\varvec{\beta }}}_i = (\beta _{1,i},\ldots ,\beta _{i-1,i})^{{}^{\mathrm{T}}}$$.

The most convenient way to compute the Bayes factor between two competing GGMs is to work with their Gaussian belief network representations.^14^ For a GGM with precision matrix ${\mathbf{W}}$, we impose a Wishart prior onto ${\mathbf{W}}$, and we represent the GGM in terms of the parameters ${\mathbf{m}}=(m_1,\ldots ,m_M)^{{}^{\mathrm{T}}}$, ${\varvec{\sigma }}^2=(\sigma _1^2,\ldots ,\sigma _M^2)^{{}^{\mathrm{T}}}$, and

$$\begin{aligned} {\mathbf{B}} = \left( \begin{matrix} 0 &{}\quad 0 &{}\quad 0 &{}\quad \ldots &{}\quad 0 \\ \beta _{1,2} &{}\quad 0 &{}\quad 0 &{}\quad \ldots &{}\quad 0 \\ \beta _{1,3} &{}\quad \beta _{2,3} &{}\quad 0 &{}\quad \ldots &{}\quad 0 \\ \vdots &{}\quad \vdots &{}\quad &{}\quad \ddots &{}\quad \vdots \\ \beta _{1,M} &{}\quad \beta _{2,M} &{}\quad \ldots &{}\quad \beta _{M- 1,M} &{}\quad 0 \\ \end{matrix} \right) \end{aligned}$$

where $$\beta _{j,i}=0$$ if $${\mathbf{W}}_{j,i}=0$$ ($$j<i$$).

Given two GGMs ${\mathcal {M}}_1$ and ${\mathcal {M}}_2$ with precision matrices ${\mathbf{W}}^1$ and ${\mathbf{W}}^2$ we represent both as Gaussian belief networks with the regression coefficient matrices ${\mathbf{B}}^k$ whose elements are given by $\beta _{j,i}^k$ ($k=1,2$). We have $\beta _{j,i}^k=0$ if ${\mathbf{W}}^k_{j,i}=0$ ($k=1,2$) and $\beta _{j,i}^1=\beta _{j,i}^2$ if $\beta _{j,i}^1,\beta _{j,i}^2\ne 0$, so that all shared non-zero regression coefficients are equal. We assume that both GGMs share the mean vector ${\mathbf{m}}$, which we assume to be known, and the conditional variances $(\sigma _i^{2})^k = \sigma _i^2$. Let ${\mathbf{B}}$ denote the matrix of all regression coefficients which are non-zero in at last one of the GGMs. The elements of ${\mathbf{B}}$ are:

56 $$\begin{aligned} \beta _{j,i} = {\left\{ \begin{array}{ll} \beta _{j,i}^1 &{} \text {if} \;\; \beta _{j,i}^1\ne 0\;\; \text {and}\;\; \beta _{j,i}^2=0 \\ \beta _{j,i}^2 &{} \text {if} \;\; \beta _{j,i}^1=0 \;\; \text {and}\;\; \beta _{j,i}^2\ne 0\\ \beta _{j,i}^1 &{} \text {if} \;\; \beta _{j,i}^1=\beta _{j,i}^2 \end{array}\right. } \end{aligned}$$

Given *n* data points ${\mathbf{x}}_1,\ldots ,{\mathbf{x}}_n$ the tempered posteriors take the form:

$$\begin{aligned} p_{\tau }({\mathbf{W}}|{\mathbf{x}}_1,\ldots ,{\mathbf{x}}_n,{\mathcal {M}}_1,{\mathcal {M}}_2) \propto \left( \prod _{w=1}^n p({\mathbf{x}}_w|{\mathbf{m}},{\mathbf{W}}^1)\right) ^{\tau } \left( \prod _{w=1}^n p({\mathbf{x}}_w|{\mathbf{m}},{\mathbf{W}}^2)\right) ^{1-\tau } p({\mathbf{W}}) \end{aligned}$$

where $\tau \in [0,1]$ and the three precision matrices ${\mathbf{W}}^1$, ${\mathbf{W}}^2$, and ${\mathbf{W}}$ can be computed with Eq. (55) from the conditional variances $\sigma _i^2$ and the regression parameters in ${\mathbf{B}}^1$, ${\mathbf{B}}^2$ and ${\mathbf{B}}$.

Sampling from the tempered posterior can be done with Metropolis–Hastings (MH) MCMC moves which we define in the space of the non-zero regression parameters in ${\mathbf{B}}$ and in the space of the logarithms of the conditional variances $\sigma _i^2$. We obtain a new candidate state ${\mathbf{B}}^{\star }$ and $\sigma ^2_{i,\star }$ by adding randomly sampled numbers to the non-zero elements of ${\mathbf{B}}$ and to $log(\sigma _i^2)$.^15^ From the new candidate matrix ${\mathbf{B}}^{\star }$ we extract the matrices ${\mathbf{B}}^{k,\star }$ ($k=1,2$) as follows: $\beta _{j,i}^{k,\star }=0$ if ${\mathbf{W}}^k_{i,j}$ is restricted to be zero and $\beta _{j,i}^{k,\star }=\beta _{j,i}^{\star }$ otherwise. The new precision matrices ${\mathbf{W}}^{\star }$, ${\mathbf{W}}^{1,\star }$, and ${\mathbf{W}}^{2,\star }$ can then be computed from ${\mathbf{B}}^{\star }$, ${\mathbf{B}}^{1,\star }$, and ${\mathbf{B}}^{2,\star }$ with Eq. (55), and the MH acceptance probability depends on the ratio of the tempered posteriors of the new precision matrix ${\mathbf{W}}^{\star }$ and the old precision matrix ${\mathbf{W}}$.

For a proof of concept we perform a simulation study: We consider the $M=7$ genes (1$\hat{=}$LHY, 2$\hat{=}$TOC1, 3$\hat{=}$PRR9, 4$\hat{=}$PRR7, 5$\hat{=}$GI, 6$\hat{=}$Y, and 7$\hat{=}$TOC1) of the Arabidopsis networks, shown in Fig. 1, and we parametrize both graphs ${\mathcal {M}}_1$ and ${\mathcal {M}}_2$ as Gaussian belief networks. We set: ${\mathbf{m}}={\mathbf{0}}$ and $\sigma _i^2=1$ for all *i*, and the non-zero regression coefficients appearing in both graphs are set to $\beta _{j,i}=1$, while the regression coefficients appearing only in the wildtype (${\mathcal {M}}_1$) are set to $\beta \in {\mathbb {R}}$. The latter coefficients correspond to the edges ’PRR9-PRR7’ ($\beta _{2,3}$) and ’PRR7-NI’ ($\beta _{3,4}$) in Fig. 1. $\beta$ is a tuning parameter for the strength of the two additional partial correlations in ${\mathcal {M}}_1$. For $\beta =0$ the partial correlations are zero and the nested mutant network ${\mathcal {M}}_2$ is the correct model. As prior on ${\mathbf{W}}$ we use a Wishart distribution with $\text {df}=10$ degrees of freedom and the identity matrix as precision matrix ${\mathbf{P}}={\mathbf{I}}_7$. We generate data sets with $n=100$ data points from ${\mathcal {M}}_1$, and we use NETI-DIFF (with 100k iterations, a sigmoidal temperature ladder and $\varepsilon =0.1$) to compute the Bayes-factors. Figure 15 shows the results. The Bayes factors are in favour of the true wildtype network (${\mathcal {M}}_1$) if the additional regression coefficients have a sufficient size ($\beta \ge 0.3$). For low values ($\beta \le 0.2$) the Bayes factor is in favour of the mutant network (${\mathcal {M}}_2$), which is actually the true network for $\beta =0$. Only for low positive values ($\beta =0.1$ and $\beta =0.2$) the wrong model is favoured over the true model. The latter can be explained by the prior. The Wishart prior with hyperparameters $\text {df}=10$ and ${\mathbf{P}}={\mathbf{I}}_7$ corresponds to 10 pseudo data points from a GGM without any non-zero partial correlations, and hence, yield a higher penalty for the wildtype netwerk ${\mathcal {M}}_1$ than for the sparser mutant network ${\mathcal {M}}_2$.

### Application to mixture models (galaxy data)

In this Appendix we show that the new method (NETI-DIFF) can also be used to compute the Bayes factor between mixture models with different numbers of mixture components. Like Friel et al. (2014) we consider the Galaxy data from Richardson and Green (1997), which contain $n=82$ measurements $y_1,\ldots ,y_{82}$ of galaxy velocities, and we compute the Bayes factor between two Bayesian Gaussian mixture models ${\mathcal {M}}_3$ with $K=3$ components and ${\mathcal {M}}_4$ with $K=4$ components. For our study we use exactly the same mixture model as Friel et al. (2014) with the same prior distributions and the same hyperparameters. A latent allocation vector ${\mathbf{z}}=(z_1,\ldots ,z_{82})^{{}^{\mathrm{T}}}$ allocates the individual data points to the *K* mixture components, where $z_i=k$ if data point $y_i$ has been allocated to component *k* ($k=1,\ldots ,K$; $i=1,\ldots ,82$). On the mixture weights $w_k:=P(z_i=k)$ we impose a Dirichlet prior:

$$\begin{aligned} (w_1,\ldots ,w_K) \sim \text {DIR}(1,\ldots ,1) \end{aligned}$$

The data points within each component *k* ($1\le k\le K$) are assumed to stem from a univariate Gaussian distribution with mean $\mu _k$ and variance $\sigma _k^2$, so that

$$\begin{aligned} y_i|(z_i=k) \sim \text {N}(\mu _k,\sigma ^2_k) \end{aligned}$$

and for $\mu _k$ and $\sigma _k^2$ we use a Gaussian prior and an Inverse-Gamma prior:

$$\begin{aligned} \mu _k \sim \text {N}(0,1000) \quad \quad \quad \sigma _k^{-2} \sim \text {GAM}(1,1) \end{aligned}$$

We define ${\varvec{\theta }}_K$ to be the set of all parameters of the mixture model ${\mathcal {M}}_K$ with *K* components:

$$\begin{aligned} {\varvec{\theta }}_K = \left\{ w_{{{K}},1},\ldots ,w_{{{K}},K},\;\mu _{{{K}},1},\ldots ,\mu _{{{K}},K},\; \sigma ^2_{{{K}},1},\ldots ,\sigma ^2_{{{K}},K} \right\} \end{aligned}$$

In the absence of limiting conditions, mixture models with different numbers of components (here: ${\mathcal {M}}_3$ and ${\mathcal {M}}_4$) do *not* share any parameters, and the tempered NETI-DIFF posteriors take the form

$$\begin{aligned} p_{\tau }({\varvec{\theta }}_3,{\varvec{\theta }}_4 |y_1,\ldots ,y_m,{\mathcal {M}}_3,{\mathcal {M}}_4)\propto & {} p(y_1,\ldots ,y_n|{\varvec{\theta }}_3)^{\tau } p(y_1,\ldots ,y_n|{\varvec{\theta }}_4)^{1-\tau }\\&p({\varvec{\theta }}_3|{\mathcal {M}}_3) p({\varvec{\theta }}_4|{\mathcal {M}}_4) \end{aligned}$$

Because of this modular form, the parameters in the sets ${\varvec{\theta }}_3$ and ${\varvec{\theta }}_4$ can be sampled by disjunct MCMC sampling steps, which either re-sample subsets of the parameters ${\tilde{{\varvec{\theta }}}}_3 \subset {\varvec{\theta }}_3$ (or ${\tilde{{\varvec{\theta }}}}_4 \subset {\varvec{\theta }}_4$) from their full conditional distributions:

$$\begin{aligned} p_{\tau }\left( \tilde{{\varvec{\theta }}_3}|\tilde{\tilde{{\varvec{\theta }}_3}}, {\varvec{\theta }}_4,y_1,\ldots ,y_m,{\mathcal {M}}_3,{\mathcal {M}}_4\right)\propto & {} p\left( y_1,\ldots ,y_n|{\varvec{\theta }}_3\right) ^{\tau } \;\;\;\;\cdot p\left( {\varvec{\theta }}_3|{\mathcal {M}}_3\right) \\ p_{\tau }\left( \tilde{{\varvec{\theta }}_4}|\tilde{\tilde{{\varvec{\theta }}_4}},{\varvec{\theta }}_3,y_1,\ldots ,y_m,{\mathcal {M}}_3,{\mathcal {M}}_4\right)\propto & {} p\left( y_1,\ldots ,y_n|{\varvec{\theta }}_4\right) ^{1-\tau } \cdot p\left( {\varvec{\theta }}_4|{\mathcal {M}}_4\right) \end{aligned}$$

where $\tilde{\tilde{{\varvec{\theta }}_K}}\cup \tilde{{\varvec{\theta }}_K} = {\varvec{\theta }}_K$, or via Metropolis Hastings sampling steps, whose acceptance probabilities are:

$$\begin{aligned} A\left( ({\varvec{\theta }}_3,{\varvec{\theta }}_4)\rightarrow ({\varvec{\theta }}_3^{\star },{\varvec{\theta }}_4)\right)= & {} \min \left\{ 1,\left( \frac{ p(y_1,\ldots ,y_n|{\varvec{\theta }}_3^{\star })}{p(y_1,\ldots ,y_n|{\varvec{\theta }}_3)}\right) ^{\tau } \;\;\;\;\cdot \frac{p({\varvec{\theta }}_3^{\star }|{\mathcal {M}}_3)}{p({\varvec{\theta }}_3|{\mathcal {M}}_3)} \cdot \text {HR}\right\} \\ A\left( ({\varvec{\theta }}_3,{\varvec{\theta }}_4)\rightarrow ({\varvec{\theta }}_3,{\varvec{\theta }}_4^{\star })\right)= & {} \min \left\{ 1,\left( \frac{p(y_1,\ldots ,y_n|{\varvec{\theta }}_4^{\star })}{p(y_1,\ldots ,y_n|{\varvec{\theta }}_4)}\right) ^{1-\tau } \cdot \frac{p({\varvec{\theta }}_4^{\star }|{\mathcal {M}}_4)}{p({\varvec{\theta }}_4|{\mathcal {M}}_4)} \cdot \text {HR}\right\} \end{aligned}$$

where HR is the move-specific Hastings ratio and the $\star$ symbol indicates a new candidate parameter set. Since these are the standard equations for power posterior sampling, as used by the thermodynamic integration (TI) approach, the adaptation of the Metropolis–Hastings and Gibbs sampling steps of the power posterior sampling scheme for TI (Friel et al. (2014)) is straightforward. At each temperature $\tau \in [0,1]$ NETI-DIFF updates the parameters in ${\varvec{\theta }}_3$ and in ${\varvec{\theta }}_4$ independently by performing the corresponding steps of the MCMC sampling scheme. The only difference is that the parameters in ${\varvec{\theta }}_4$ are subject to the complementary temperature $1-\tau$ rather than $\tau$, and we therefore implement NETI-DIFF with the sigmoid inverse temperature ladder from Sect. 3.6. Moreover, we also take into account that NETI-DIFF has to perform twice as many sampling steps as TI, since NETI-DIFF re-samples the parameters of both models ${\mathcal {M}}_3$ and ${\mathcal {M}}_4$ within each iteration. Thus, NETI-DIFF iterations are approximately double as expensive as TI iterations, and we can perform only 50% of the total number of iterations $N_{iter}$ with NETI-DIFF.

In our empirical study we compare the performance of NETI-DIFF with TI-standard and TI-optimal, and we implement both TI approaches with 100 discretisation points. We compute the Bayes factor between the mixture models ${\mathcal {M}}_3$ and ${\mathcal {M}}_4$ based on $N_{iter}=1000k$ and $N_{iter}=2000k$ iterations.^16^ The results of our study are shown in Fig. 16. It can be seen that there are no significant differences between the performances. The NETI-DIFF estimates appear to be minimally less biased than the TI estimates, but on the other hand the NETI-DIFF estimates have a slightly increased standard deviation. This finding, that NETI-DIFF does not lead to any improvement over the standard TI approach, is not surprising: Due to the fact that the two mixture models do not have any parameters in common, targeting the Bayes factor directly cannot have any advantages. For models with disjunct parameter spaces NETI-DIFF effectively just corresponds to two simultaneously performed but independent non-equilibrium thermodynamic integration (NETI) approaches, where one model is subject to the complementary temperature transition from $\tau =1$ to $\tau =0$. Targeting the Bayes factor directly, as described in Sect. 3.3, can only lead to an improvement if the two models share parameters. In the direct transition paths between the two model posteriors, only those shared parameters constantly appear with the inverse temperature 1 and do not undergo any temperature transitions (i.e. they are excluded from the annealing process). All non-shared parameters have to undergo the transitions from $\tau =0$ to $\tau =1$ or from $\tau =1$ to $\tau =0$, respectively.

### Full conditional distributions of variance parameters

For linear models where the variance parameter $\sigma ^2$ in Eq. (20) in Sect. 3.5 is not known, a prior distribution has to be imposed on $\sigma ^2$. A common choice is the conjugate Inverse-Gamma distribution with hyperparameters *a* / 2 and *b* / 2, symbolically $\sigma ^{-2}\sim \text {GAM}(\frac{a}{2},\frac{b}{2})$. The tempered full conditional distribution of $\sigma ^{-2}$ is then of closed-form and can be derived as follows:

$$\begin{aligned} p_{\tau }(\sigma ^{-2}|D,{\varvec{\theta }},{\mathcal {M}}_1,{\mathcal {M}}_2)\propto & {} p(\mathbf{y}|{\varvec{\theta }},\sigma ^2,{\mathcal {M}}_2)^{\tau } \cdot p(\mathbf{y}|{\varvec{\theta }},\sigma ^2,{\mathcal {M}}_1)^{1-\tau } \cdot p({\varvec{\theta }}|\sigma ^2,{\mathcal {M}}_1,{\mathcal {M}}_2) \cdot p(\sigma ^2)\\\propto & {} N_n(\mathbf{{D}}^{(2)}{\varvec{\theta }},\sigma ^2{\mathbf{I}})^{\tau } \cdot N_n(\mathbf{{D}}^{(1)}{\varvec{\theta }},\sigma ^2{\mathbf{I}})^{1-\tau } \cdot N_p(\varvec{\mu }_0,\sigma ^2\delta ^2 {\mathbf{I}}) \cdot GAM(\sigma ^{-2}) \\\propto & {} \left( \frac{1}{\sigma ^2}\right) ^{\tau \cdot \frac{ n}{2}} \exp \left( -\frac{1}{2\sigma ^2} \cdot \left[ \mathbf{{D}}^{(1)}{\varvec{\theta }}-\mathbf{y}\right] ^{{}^{\top }} \left[ \mathbf{{D}}^{(1)}{\varvec{\theta }}-\mathbf{y}\right] \cdot (1-\tau ) \right) \cdot \\&\left( \frac{1}{\sigma ^2}\right) ^{(1-\tau ) \cdot \frac{ n}{2}} \exp \left( -\frac{1}{2\sigma ^2} \cdot \left[ \mathbf{{D}}^{(2)}{\varvec{\theta }}-\mathbf{y}\right] ^{{}^{\top }} \left[ \mathbf{{D}}^{(2)}{\varvec{\theta }}-\mathbf{y}\right] \cdot \tau \right) \cdot \\&\left( \frac{1}{\sigma ^2}\right) ^{\frac{ p}{2}} \exp \left( -\frac{1}{2\sigma ^2\delta ^2} \cdot [{\varvec{\theta }}-\varvec{\mu }_0]^{{}^{\top }} [{\varvec{\theta }}-\varvec{\mu }_0] \right) \cdot \\&\left( \sigma ^{-2}\right) ^{a/2-1} \exp \left( -\sigma ^{-2} \cdot \frac{b}{2} \right) \\= & {} \left( \sigma ^{-2}\right) ^{{\tilde{a}} -1} \cdot \exp \left( -\sigma ^{-2} \cdot {\tilde{b}} \right) \end{aligned}$$

where *p* is the length of the regression coefficient vector ${\varvec{\theta }}$ and

$$\begin{aligned} {\tilde{a}}= & {} \frac{1}{2} (a+n+p) \\ {\tilde{b}}= & {} \frac{1}{2} \left( b+ (1-\tau ) \left[ \mathbf{{D}}^{(1)}{\varvec{\theta }}-\mathbf{y}\right] ^{{}^{\top }} \left[ \mathbf{{D}}^{(1)}{\varvec{\theta }}-\mathbf{y}\right] \right. \\&\qquad \left. + \tau \left[ \mathbf{{D}}^{(2)}{\varvec{\theta }}-\mathbf{y}\right] ^{{}^{\top }} \left[ \mathbf{{D}}^{(2)}{\varvec{\theta }}-\mathbf{y}\right] + \delta ^{-2} [{\varvec{\theta }}-\varvec{\mu }_0]^{{}^{\top }} [{\varvec{\theta }}-\varvec{\mu }_0] \right) \end{aligned}$$

Comparing this with the identity:

$$\begin{aligned} \text {GAM}(\sigma ^{-2}|{\tilde{a}},{\tilde{b}}) = \frac{{\tilde{b}}^{{\tilde{a}}}}{\varGamma ({\tilde{a}})} \cdot \left( \sigma ^{-2}\right) ^{{\tilde{a}}-1} \exp \{- \sigma ^{-2}\cdot {\tilde{b}} \} \propto \left( \sigma ^{-2}\right) ^{{\tilde{a}}-1} \exp \{- \sigma ^{-2}\cdot {\tilde{b}} \} \end{aligned}$$

we get the full conditional distribution

$$\begin{aligned} \sigma ^{-2}|(D,{\varvec{\theta }},{\mathcal {M}}_1,{\mathcal {M}}_2,\tau )\sim \text {GAM}({\tilde{a}},{\tilde{b}}) \end{aligned}$$

Hence, we can also sample $\sigma ^{-2}$ directly from the tempered full conditional distribution in a Gibbs sampling scheme, and $\sigma ^2 = 1/\sigma ^{-2}$.

**Table 3:** Runtimes in seconds for different data sets and 1,00,000 iterations.

	*n*	NETI-DIFF	TI-standard	TI-optimal
Radiata pine	42	81.6 (4.1)	85.6 (4.4)	85.6 (4.4)
Pima Indians	532	9.3 (0.1)	9.5 (0.3)	10.3 (0.1)
Radiocarbon	343	19.5 (0.5)	–	35.4 (0.8)
Biopepa	143	54.1 (1.2)	53.6 (2.0)	58.7 (2.5)

The data size is given by *n* observations. The number of variables can differ depending on the model. Time values are averages over 10 runs and different models on an Intel Core i7 6700 HQ processor. The standard deviations are indicated in brackets. The runtime for Biopepa is for a single response out of seven possible responses

### Computational run times and convergence diagnostics

It is important to assess the convergence of the NETI simulations accurately. However, conventional convergence diagnostics for MCMC, like the Gelman–Rubin potential scale reduction factor, are not applicable here. The reason is that the combination of the NETI scheme, described in Sect. 3.2, and the new thermodynamic integration path, described in Sect. 3.3, continuously transform one model into another via a series of non-equilibrium configurations. We need to point out that any samples taken during this transformation are of no interest in themselves; the only quantity of interest is the log Bayes factor, computed according to Eq. (18). The estimate of the log Bayes factor from Eq. (18) is a random variable that is subject to the intrinsic stochasticity of the MCMC sampler. A natural convergence diagnostic is the variance of this estimator: for an infinite simulation time, the variance should go to zero as the estimate should not depend on the particular idiosyncrasies of any MCMC trajectory. We have investigated this conjecture in Figs. 6, 7, 11c, d and 13a. Since Figs. 11c, d and 13a provide average variances over five independent data instantiations, we have included Fig. 17 that shows the individual variances for each data set separately. All these figures demonstrate that the variance approaches zero as the simulation time, regarding the number of MCMC steps, is increased. Figure 6 quantifies the improvement in convergence that the proposed method achieves over the established schemes, in the form of a faster decrease of the variance with increasing simulation times.

The figures mentioned above, e.g. Figs. 6 and 13a, monitor convergence in terms of iteration numbers. For a fair comparison between different methods, we also need to take into consideration the computational costs per iteration shown in Table 3: The computational run times of the three algorithms compared are approximately equal; if there is any difference at all, it appears to be in favour of the proposed NETI scheme. From this, we can conclude that monitoring inference uncertainty as a function of MCMC iteration numbers, as carried out throughout our paper, provides an appropriate quantification of computational complexity.

## Footnotes

1 Note that in the regression example presented by Neal (2001), where the objective is model selection between a Gaussian and a Cauchy distribution for the noise, the log marginal likelihoods are computed separately with annealed importance sampling, and then combined to produce the log Bayes factor. Unlike the scheme proposed in the present article, the log Bayes factor is *not* targeted directly.

2
$$\int _a^b f(x) dx = {(b-a)} \frac{f(b)+f(a)}{2} - \frac{(b-a)^3}{12} f''(c)$$ for some $$c\in [a,b]$$.

3 Note that *K* can be set equal to the total number of MCMC iterations $N_{iter}$, which otherwise would have to be subdivided onto *K* discretisation points.

4 The extrapolation scheme proposed by Schlitter and Husmeier (1992) can reduce actual computation time by parallelisation, but this was not an issue for the simulations carried out in the present work.

5 For uneven $N_{iter}$, we fix one point at $\tau =0.5$ and apply the procedure to the remaining $N_{iter}-1$ points.

6 Unlike the prior in Eq. (20), the prior from Friel et al. (2014) uses fixed variances in Eq. (31).

7 Conditional on its Markov blanket, a node is independent of the rest of the graph; so the Markov blanket shields a node from the remaining graph. The Markov blanket of a node is the set of nodes in the graph that consists of the parents, the co-parents, and the children. In a graph A $\rightarrow$ B $\leftarrow$ C, we have: A is a parent of B (it has a directed edge from A to B), B is a child of both A and C, C is a co-parent of A, and A is a co-parent of C.

8 Note that there is a typo in Eq. (17) of Friel et al. (2014); $t = \frac{{\hat{f}}_{k+1} - {\hat{f}}_{k} + {\hat{f}}_{k}{\hat{V}}_{k} -{\hat{f}}_{k+1}{\hat{V}}_{k+1} }{{\hat{V}}_{k}-{\hat{V}}_{k+1}}$ must read: $t = \frac{{\hat{f}}_{k+1} - {\hat{f}}_{k} + t_{k}{\hat{V}}_{k} -t_{k+1}{\hat{V}}_{k+1} }{{\hat{V}}_{k}-{\hat{V}}_{k+1}}.$

9 Due to the non-equilibrium nature of NETI, not discarding any burn-in phase made little difference to the results.

10 This is a consequence of the fact that due to the non-nested structure of the models, there is always a parameter for which the transition effectively moves from the posterior to the prior, rendering the power law of Eq. (8) suboptimal.

11 NETI-DIFF is unaffected because it does not depend on the *K* number of discretization points of the integral as the classical TI does. Instead, it continuously transforms one model into the other.

12 These results were obtained from the first two data sets in the first column of Table 1.

13
Berger and Delampady (1987) study the Bayesian test problem: ${\mathcal {H}}_0: \theta =\theta _0$ vs. ${\mathcal {H}}_1: \theta \ne \theta _0$, where $\theta$ is a continuous parameter. In Berger and Delampady (1987) the denominator of the Bayes factor *B* in Eq. (3) is given by: $P({\mathcal {D}}|{\mathcal {H}}_1)=\int p({\mathcal {D}}|\theta ) g(\theta ) d\theta$, where the prior *g*(.) and the integral are over all parameters belonging to ${\mathcal {H}}_1$. Here we can think of the test: ${\mathcal {H}}_0: {\mathcal {M}}={\mathcal {M}}_1$ vs. ${\mathcal {H}}_1: {\mathcal {M}}\ne {\mathcal {M}}_1$. With the partition theorem we get for the joint probability: $p(D,{\mathcal {H}}_1)=p(D,\{{\mathcal {M}}_2\cup \ldots \cup {\mathcal {M}}_m\})= \sum _{j=2}^m p(D,{\mathcal {M}}_j) = \sum _{j=2}^m p(D|{\mathcal {M}}_j) \cdot \pi _j$, and hence we have for the denominator of our Bayes factor: $p(D|{\mathcal {H}}_1)=\frac{p(D,{\mathcal {H}}_1)}{1-\pi _1}=\sum _{j=2}^m p(D|{\mathcal {M}}_j)\cdot g({\mathcal {M}}_j)$.
